# PathoGraph: A Graph-Based Method for Standardized Representation of Pathology Knowledge

**DOI:** 10.1038/s41597-025-04906-z

**Published:** 2025-05-27

**Authors:** Peiliang Lou, Yuxin Dong, Caixia Ding, Chunbao Wang, Ruifeng Guo, XiaoBo Pang, Chen Wang, Chen Li

**Affiliations:** 1 School of Computer Science and Technology, Xi’an Jiaotong University, Xi’an, Shaanxi 710049 China; 2 Department of Pathology, Shaanxi Provincial Tumor Hospital, Xi’an Jiaotong University, 309 Yanta West Road, Xi’an, Shaanxi China; 3 Department of Pathology, The First Affiliated Hospital of Xi’an Jiaotong University, 277 West Yanta Road, Xi’an, Shaanxi China; 4 Division of Anatomic Pathology, Department of Laboratory Medicine and Pathology, Mayo Clinic, Jacksonville, Florida USA; 5 Division of Computational Biology, Mayo Clinic, Rochester, MN USA; 6 National Engineering Lab for Big Data Analytics, Xi’an Jiaotong University, Xi’an, Shaanxi China

**Keywords:** Cancer, Data integration

## Abstract

Pathology data, primarily consisting of slides and diagnostic reports, inherently contain knowledge that is pivotal for advancing data-driven biomedical research and clinical practice. However, the hidden and fragmented nature of this knowledge across various data modalities not only hinders its computational utilization, but also impedes the effective integration of AI technologies within the domain of pathology. To systematically organize pathology knowledge for its computational use, we propose PathoGraph, a knowledge representation method that describes pathology knowledge in a graph-based format. PathoGraph can represent: (1) pathological entities’ types and morphological features; (2) the composition, spatial arrangements, and dynamic behaviors associated with pathological phenotypes; and (3) the differential diagnostic approaches used by pathologists. By applying PathoGraph to neoplastic diseases, we illustrate its ability to comprehensively and structurally capture multi-scale disease characteristics alongside pathologists’ expertise. Furthermore, we validate its computational utility by demonstrating the feasibility of large-scale automated PathoGraph construction, showing performance improvements in downstream deep learning tasks, and presenting two illustrative use cases that highlight its clinical potential. We believe PathoGraph opens new avenues for AI-driven advances in the field of pathology.

## Introduction

The field of pathology has yielded vast data resources, which contain meaningful knowledge for biomedical research and clinical practice. Driven by Artificial Intelligence (AI), frontier research and applications related to pathology emerge, including computational oncology^1^, deep phenotyping^2,3^, and automatic pathological diagnosis^4^ etc. These advanced directions rely on computational use of pathology knowledge within multi-modal pathology data. However, pathology knowledge is disorganized, fragmented and hidden among images and texts. Developing a formalized and standardized way to represent pathology knowledge, making it accessible to computational use, could largely improve the application of AI in pathology.

**Fig. 1:**
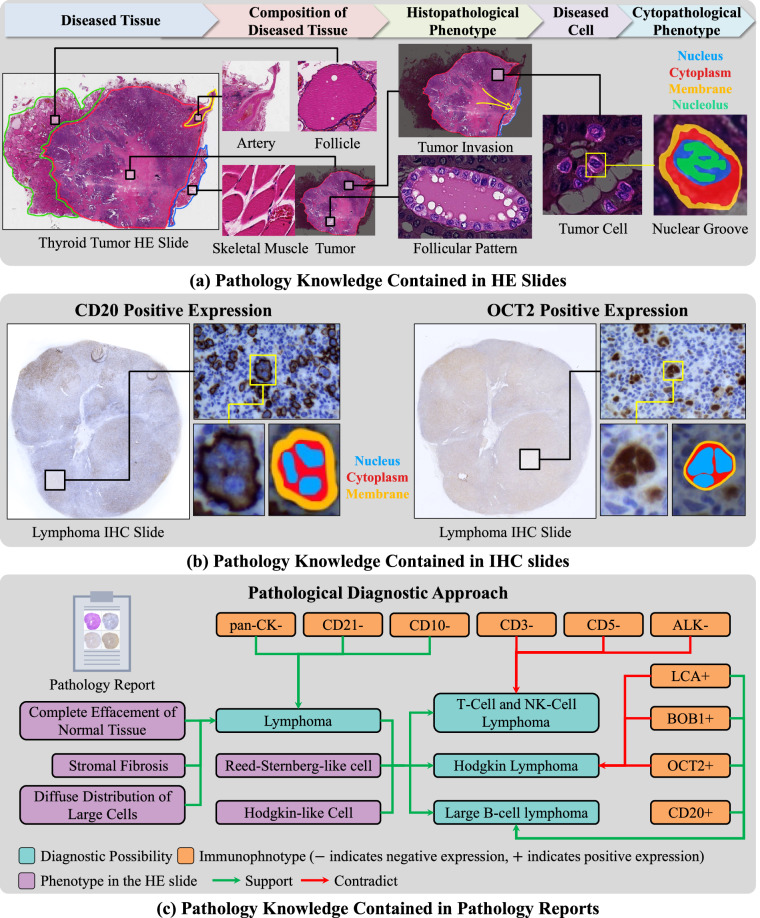
Pathology knowledge contained in different modalities of pathology data. **(a)** The compositions of the diseased tissue and phenotypes in a thyroid carcinoma hematoxylin and eosin-stained (HE) slide, reflecting the characteristics of the disease at the tissue and cellular level. **(b)** Two lymphoma immunohistochemistry (IHC) slides stained for the antigens of CD20 and OCT2, respectively. The brown areas in the slides are the stained antigens, where CD20 are stained in the tumor cell membranes (i.e. CD20 resides in the membranes) and OCT2 are stained in the nuclei. The normal cells, which do not stain for these antigens, appear blue. The immunophenotypes of CD20 and OCT2 reflect the characteristics of the disease at the protein level. **(c)** The schematic representation of a pathologist's diagnostic process to subtype a patient with lymphoma obtained from a pathology report. Firstly, the phenotypes in the HE slide lead to an initial diagnosis that this tumor is a lymphoma. Secondly, the pathologist considers three possible subtypes of lymphoma aligning with these phenotypes. To differentiate these diagnostic possibilities, additional IHC results are introduced, such as CD3, CD5, etc. The IHC results support large B-cell lymphoma and contradict the other subtypes, leading to its establishment as the final diagnosis.

Pathology knowledge refers to information about pathological changes related to diseases, including the composition of diseased tissues, their pathological characteristics and relationships at the protein, cellular, and tissue levels etc. It also encompasses diagnostic expertise of pathologists regarding these changes. Pathology knowledge is contained in pathology data of different modalities. Table 1 outlines three widely-applied data modalities and summarizes the pathology knowledge they contain. Figure 1 further elucidates the knowledge described in Table 1 by providing several examples. Pathology knowledge contained in pathology data is of great value in addressing challenges in biomedical research and clinical practice by using data-driven approaches. Recently, the computational use of pathology knowledge based on deep learning techniques has generated novel insights related to cancer and facilitated automated pathologist-like diagnostic process^5^.

**Table 1:** Pathology Knowledge Contained in Three Widely-applied Pathology Data Modalities.

Pathology Data	Pathology Knowledge Contained
Hematoxylin and Eosin-stained Slide (a pathological slide created by staining cells and tissues in a diseased tissue with Hematoxylin and Eosin dyes)	**Composition of diseased tissue:** Residual normal tissues and cells either surrounding or interspersed within diseased tissues, along with diseased cells and their intracellular components, as well as chemical substances produced or stored by diseased cells, among others.
	**Histopathological Phenotype:** The characteristics of diseased tissues at the tissue level, including the growth patterns and biological behaviors (such as compression, invasion, and infiltration towards normal tissue), and the spatial arrangement patterns of diseased cell populations (such as acinar arrangement, papillary arrangement, etc.).
	**Cytopathological Phenotype:** The characteristics of diseased tissues at the cellular level, including changes in morphology and distribution location of diseased cell as well as its components (such as irregular nuclear shapes, alterations in cell size, etc.).
Immunohistochemistry Slide (a pathological slide created by staining antigens in a diseased tissue with one or more antibodies)	**Immunophenotype:** The characteristics of diseased tissues at the protein level, including changes in the types of proteins carried by diseased cells, alterations in proteins’ distribution locations within diseased cells, and variations in their quantity.
Pathology Report (a report recording the diagnosis process and results of a pathologist on a diseased tissue)	**Pathological Diagnostic Approach:** This refers to the systematic thought process of pathologists, which involves analyzing phenotypic characteristics, using phenotypes as a basis for diagnosis, and considering and excluding multiple diagnostic possibilities to ultimately arrive at the definitive diagnostic conclusion.

However, pathology knowledge is hidden within both textual data and images, which is not conducive to its computational utilization. Taking a pathological slide as an example, although it provides detailed insights into the pathological aspects of a tissue sample, as Fig. 1a shows, such information is embedded within the pixels, which requires expert interpretation to be fully apparent and amenable to analysis. As for the textual data, words and sentences can be obscure when describing pathological concepts or phenomena. For example, malignant cells are usually described as “atypical” in biomedical literatures, while only professionals understand what qualities “atypical” refers to. As a result, computational methods may struggle to fully understand and analyze pathology data, as pathology knowledge is implicitly captured in the data modalities, making it less accessible for computational analysis.

**Fig. 2:**
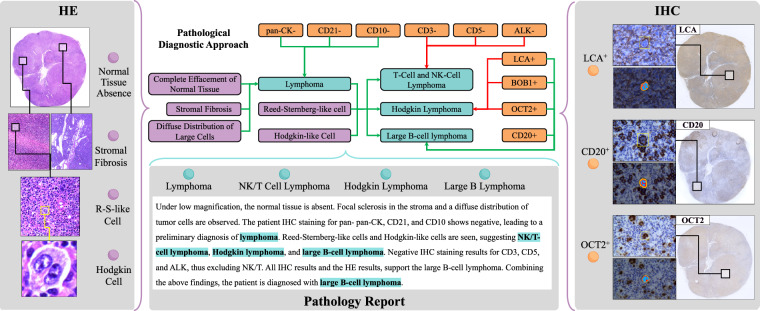
The schematic representation of the fragmentation of pathology knowledge across various pathology data modalities, with the diagnostic process depicted in Fig. 1c as an example.

Additionally, pathology knowledge is fragmented across data modalities, which also impedes its computational utilization. For instance, Fig. 1c illustrates the key aspects of a pathologist’s diagnostic process, which involves not only the identification and evaluation of microscopic findings from pathology slides, but also the differential diagnosis that correlates these findings with diagnostic possibilities, and narrows down these possibilities to confirm the final one. However, the evidential phenotypes and the diagnostic insights that a pathologist derives are fragmented across various data sources. Figure 2 depicts how the elements of the diagnostic process, as outlined in Fig. 1c, are scattered as incomplete and disconnected pieces across different datasets. The fragmented nature of pathology knowledge hinders its suitability for computational analysis, thereby preventing AI methods from fully leveraging the expertise encapsulated in pathology data.

To facilitate AI-based pathology practice, it requires a formalized representation of pathology knowledge hidden within and fragmented across pathology data, making it more accessible, organized and usable for computational analysis. In this paper, we propose a method for representing pathology knowledge in a graph structure, namely PathoGraph. Specifically, PathoGraph defines three graphs, each representing different aspects: 1) the types and morphological characteristics of pathological entities across biological scales; 2) the components of pathological phenotypes as well as their spatial arrangement and dynamic behaviors; 3) the evaluation and differential diagnostic processes of pathologists for patients. Furthermore, to provide a standardized format for PathoGraph in line with FAIR principles^6^, we develop PathoGraph into a markup language called PathoML. We illustrate PathoGraph’s utility by applying it to represent pathology knowledge in neoplastic diseases, and we evaluate how PathoGraph enhances the computational use of pathology knowledge through a series of technical validations, including (1) feasibility of large-scale automated PathoGraph construction, (2) performance improvements in AI models, and (3) potential clinical applications. Collectively, we believe our efforts lay a critical foundation for constructing pathology knowledge graphs, thereby advancing AI-driven pathology practice.

## Results

### Overview of PathoGraph

PathoGraph represents pathology knowledge contained in multimodal pathology data in the form of a graph *G* = (${\mathscr{V}}$, ${\mathscr{E}}$). A graph representation of information consists of a set of nodes ${\mathscr{V}}$ connected by a set of edges ${\mathscr{E}}$, where each node describes a real-world entity and each edge describes a relationship between them; together, nodes and edges represent the meaning of the information.

PathoGraph focuses on pathology knowledge contained in HE slides, IHC slides, and pathology reports, which are three of the most widely applied and abundant information resources in the field of pathology. These modalities contain not only multi-scaled characteristics related to diseases, but also the diagnostic expertise of pathologists, which constitute the core content of pathology knowledge, as demonstrated by Table 1 and Fig. 1. To effectively represent such knowledge information, PathoGraph defines three graphs: (1) Pathology Entity Graph, which maps out the basic entities in pathology; (2) Pathology Phenotype Graph, illustrating various pathological phenotypes; and (3) Pathology Diagnosis Graph, depicting diagnostic processes of pathologists. In order to link together different contexts of a disease, these graphs are organized into three layers, with associations across these layers. For example, a cell’s characteristics represented in the Pathology Entity Graph (one layer) could be associated with its manifestation in the Pathology Phenotype Graph (another layer), illustrating how individual cellular features contribute to the overall pathological phenotype.

In the following sections, we introduce the three graphs respectively by illustrating their nodes and relationships, and providing their formal definitions. To ensure precision and clarity in our description, we will employ the notations and formulations of graph theory throughout the paper. Furthermore, we introduce PathoML, which is an OWL-based mark-up language for storing and exchange of PathoGraph representations.

### Pathology entity graph

Pathology Entity Graph consists of three types of nodes: pathological entity, pathological feature, and quantitative parameter, as well as their relationships.

Pathological entities refer to biological structures that constitute diseased tissues, which can be observed microscopically on pathological slides. Pathological entities encompass both diseased structures and, in certain cases, the remaining normal biological components. These entities span multiple biological scales, including molecules, cells, and tissues. There exists a hierarchical composition relationship among different pathological entities. For example, cells are components of a tissue, and a cell is composed of cell membranes, cytoplasm, and nuclei, which are further made up of molecules. Supplementary Table 1 provides three examples of pathological entities and their hierarchical component structures.

Pathological features refer to characteristics of pathological entities that could change as diseases progress, including both morphological and functional traits. For example, diseased cells and normal cells differ in their shape and size. In addition, cancer cells show differences in cytoplasmic acid-base affinity and proliferative activity based on their level of malignancy. Supplementary Table 2 enumerates various pathological features of tumor cells and their components, which often undergo changes as the tumor progresses.

Quantitative parameters refer to measurable quantities that can quantitatively assess the extent of changes in pathological features. Compared to using descriptive terms such as “significantly enlarged” “pronounced” or “minimal”, quantitative parameters can describe the changes in a pathological feature more objectively. For example, a cell nucleus, which is normally round in shape, undergoes irregular shape changes in a diseased state. These changes can be quantified and correlated with morphological parameters such as shape factor and roundness, which are obtainable from pathological slides using morphometric techniques. Supplementary Table 3 lists some examples of quantitative parameters.

Based on these three fundamental elements, the definition of Pathology Entity Graph is provided as follows:

**Definition 1 Pathology Entity Graph**: For a pathological entity $a\in A$, its graph representation is denoted as ${{\mathscr{G}}}_{a}=(\{{a\}\cup E}_{a}\cup {C}_{a}\cup {Q}_{a},{R}_{{ee}}\cup {R}_{{ec}}\cup {R}_{{cq}})$, where

- ${E}_{a}=\{{e}_{1},{e}_{2},\ldots ,{e}_{N}\}$ indicates the entity’s components;
- ${C}_{a}=\{{c}_{1},{c}_{2},\ldots ,{c}_{K}\}$ indicates the pathological features of this entity and its components;
- ${Q}_{a}=\{{q}_{1},{q}_{2},\ldots ,{q}_{T}\}$ indicates a group of quantitative parameters;
- ${R}_{{ee}}\subseteq (\{a\}\cup {E}_{a})\times {E}_{a}$ indicates the part-whole relationships between the entity and its components, as well as the relationships among the components themselves;
- ${R}_{{ec}}\subseteq (\{a\}\cup {E}_{a})\times {C}_{a}$ indicates the corresponding relationships between the pathological features and both the entity and its individual components, signifying that these features are inherent to the entity or to its components;
- ${R}_{{cq}}\subseteq {C}_{a}\times {Q}_{a}$ indicates the corresponding relationships between the pathological features and the quantitative parameters, signifying that these parameters quantitatively characterize the pathological features.

A schematic overview of ${{\mathscr{G}}}_{a}$ is provided in Supplementary Figure 1a.

### Pathology phenotype graph

Pathological phenotypes refer to specific changes that can be used to distinguish different pathological conditions and reflect the underlying nature of diseases. Pathological phenotypes are traditionally classified into histopathological phenotypes, cytopathological phenotypes, and immunophenotypes. However, for the purpose of graphical representation, we have redefined these phenotypes from the perspective of graph theory, categorizing them into three types: single-cell phenotype, multi-cell phenotype, and quantitative phenotypic indicator. For each of these three types, we propose a corresponding graph structure. Collectively, these graphs form Pathology Phenotype Graph, each serving as a subgraph.

#### Single-cell phenotype

Single-cell phenotypes refer to phenotypic manifestations observed in an individual cell, which may include changes in cell morphology such as alterations in cytoplasmic structure and nuclear shape, or variations in protein expression levels within the cell. Supplementary Table 4 lists some examples of single-cell phenotypes. The key to objectively characterizing such a phenotype is to identify the cellular components that have undergone pathological alterations, specify their pathological features that have changed, and quantitatively describe the extent of the changes, as well as the cell’s hierarchical composition structure. In view of this, we provide the definition for the Single-Cell Phenotype Graph as follows:

**Definition 2 Single-Cell Phenotype Graph**: For a single-cell phenotype ${sp}\in P$, its graph representation is denoted as ${{\mathscr{G}}}_{{sp}}=(\{{sp}\}\cup {a}_{{sp}},{R}_{{pa}})$, where

- ${a}_{{sp}}$ indicates the cell involved in this phenotype;
- ${R}_{{pa}}\subseteq \{{sp}\}\times {a}_{{sp}}$ indicates the corresponding relationship between the phenotype and the cell;

**Extended Definition 2** By leveraging Pathology Entity Graph, we can expand the graph representation of a single-cell phenotype. The expanded graph is denoted as ${{\mathscr{G}}}_{{sp}}^{* }=(\{{sp}\}\cup {{\mathscr{G}}}_{{a}_{{sp}}},{R}_{{pa}})$, where ${{\mathscr{G}}}_{{a}_{{sp}}}$ is the Pathology Entity Graph of ${a}_{{sp}}$. It enriches ${{\mathscr{G}}}_{{sp}}^{* }$ by integrating the individual cell’s components, pathological features, and quantitative parameters associated with the single-cell phenotype.

A schematic overview of ${{\mathscr{G}}}_{{sp}}^{* }$ is provided in Supplementary Figure 1b.

#### Multi-Cell Phenotype

Multi-cell phenotypes refer to phenotypic manifestations composed of several cells and other microscopically observable pathological entities such as tissues, bio-molecules (e.g mucin, acid and starch) or anatomical structures (e.g. cavity). The characteristics of such a phenotype include not only the pathological features of each individual entity, but also the spatial arrangement pattern and the dynamic behaviors (e.g invasion, extension) among the group of entities, which emerge from their interactions. Supplementary Table 5 lists some examples of multi-cell phenotypes. To fully reveal the characteristics of a multi-entity pathological phenotype, we define Multi-Cell Phenotype Graph as follows:

**Definition 3 Multi-Cell Phenotype Graph**: For a multi-cell phenotype ${mp}\in P$, its graph representation is denoted as ${{\mathscr{G}}}_{{mp}}=({\{{mp}\}\cup E}_{{mp}}\cup \Omega ,{R}_{{pa}}\cup {R}_{{sb}})$, where

- ${E}_{{mp}}=\{{a}_{1},{a}_{2},\ldots ,{a}_{M}\}$ indicates a group of pathological entities composing the phenotype;
- $\Omega =\{{S}_{{mp}},{D}_{{mp}}\}$ indicates the union of notations representing the spatial arrangement relationships and dynamic behaviors;
- ${R}_{{pa}}\subseteq \{{mp}\}\times {E}_{{mp}}$ indicates the corresoponding relationship between the phenotype and the involved pathological entities;
- ${R}_{{sb}}\subseteq {E}_{{mp}}\times \Omega \times {E}_{mp}^{{\prime} }$ indicates the relationships reflecting the spatial arrangement and dynamic behaviors of the pathological entities within the phenotype;

**Extended Definition 3** Similarly, we can expand the graph representation of a multi-cell phenotype by using Pathology Entity Graph. The expanded graph is denoted as ${{\mathscr{G}}}_{{mp}}^{* }=(\{{mp}\}\cup {G}_{{E}_{{mp}}}\cup \Omega ,{R}_{{pa}}\cup {R}_{{sb}})$, where ${G}_{{E}_{mp}}={\{{{\mathscr{G}}}_{{a}_{i}}\}}_{{a}_{i}\in {E}_{{mp}}}$ is a group of Pathology Entity Graphs, with each ${{\mathscr{G}}}_{{a}_{i}}$ representing the characteristics of a composing pathological entity ${a}_{i}$ in the phenotype.

A schematic overview of ${{\mathscr{G}}}_{{mp}}^{* }$ is provided in Supplementary Figure 1b.

#### Quantitative phenotypic indicators

To minimize variability and ambiguity in pathologists’ interpretations of pathological phenotypes, experts in pathology and professional organizations have recommended some quantitative phenotypic indicators. These indicators are crucial for diagnosis and also enhance the precision in characterizing pathological phenotypes. Supplementary Table 6 lists some examples of quantitative phenotypic indicators. The numerical values of quantitative phenotypic indicators are calculated based on quantitative parameters of specific pathological features associated with certain pathological entities, through pre-defined mathematical functions (e.g. TSR and TILs) or visual assessment (HER2 expression), depending on the context. Based on this, we provide the definition for the Quantitative Indicator Graph as follows:

**Definition 4 Quantitative Indicator Graph**: For a quantitative indicator ${qi}\in P$, its graph representation is denoted as ${{\mathscr{G}}}_{{qi}}=({\left\{{qi}\right\}\cup Q}_{{qi}}\cup \{v\},{R}_{{qq}}\cup {R}_{{qv}}\cup {f}_{{qv}})$, where

- ${Q}_{{qi}}=\{{q}_{1},{q}_{2},\ldots ,{q}_{T}\}$ indicates the group of quantitative parameters used in ${qi}$‘s calculation;
- $v$ belongs to a set of real numbers ${\mathbb{R}}$, and indicates the numerical value of ${qi}$;
- ${R}_{{qq}}=\{({qi},{Q}_{{qi}})\}$ indicates the corresponding relationship between ${qi}$ and its quantitative parameters;
- ${R}_{{qv}}=\{({qm},v)\}$ indicates the corresponding relationship between ${qi}$ and its numerical value;
- ${f}_{{qv}}:{Q}_{{qi}}{\mathbb{\to }}{\mathbb{R}}$ indicates a function from ${Q}_{{qi}}$ to ${\mathbb{R}}$, which is the mathematical formula for calculating ${qi}$'s value.

A schematic overview of ${{\mathscr{G}}}_{{qi}}$ is provided in Supplementary Figure 1b.

### Pathology diagnosis graph

Pathology Diagnosis Graph represents the evaluation and diagnostic processes of pathologists for patients based on their pathological slides. Such graph consists of several types of nodes: diagnostic item, diagnostic process, diagnostic stage, diagnostic possibilities and outcome, and diagnostic feature.

Diagnostic items are key data elements of pathology reports to which pathologists should deliver definitive diagnostic outcomes. These items are devised by hospitals’ pathology departments, based on comprehensive disease reporting requirements and patient care optimization, and are often guided by professional standards. For instance, the College of American Pathologists (CAP) cancer protocol^7^ stipulates diagnostic items for renal tumors, which encompass the histologic grading of tumor cells^8^ and the TNM staging^9^, among others.

Given the multiple diagnostic possibilities associated with each diagnostic item, the pathologist is required to undertake a diagnostic process to ascertain the most appropriate one, referred to as the diagnostic outcome. Such process can be divided into different stages including preliminary diagnosis, further diagnosis, and final diagnosis, each contributing progressively to a more refined and accurate diagnosis. A diagnostic stage mainly includes three steps: 1) examining the patient’s pathology data, 2) considering all diagnostic possibilities, 3) differentiating among these possibilities to exclude incorrect ones. The pathologist iteratively performs these steps at each stage, proceeds with further diagnostic evaluations, and gradually narrows down the range of possibilities. This process continues until the most accurate possibility is pinpointed. When making a diagnostic decision, the pathologist primarily considers the pathological phenotypes observed microscopically on pathological slides, known as diagnostic features. The diagnostic possibility that are supported by most diagnostic features are established as the diagnostic outcome while others are excluded.

Based on the above abstraction of the diagnostic process, we provide the definition for Pathology Diagnosis Graph as follows:

**Definition 5 Pathology Diagnosis Graph**: For a diagnostic process ${dp}\in {DP}$, its graph representation is denoted as ${{\mathscr{G}}}_{{dp}}=({DS},{R}_{{ss}},{R}_{{ds}})$, where

- ${DS}={\{{{DS}}_{i}\}}_{{s}_{i}\in {S}_{{dp}}}$, where ${S}_{{dp}}=\{{s}_{1},{s}_{2},\ldots ,{s}_{N}\}$ indicates the group of diagnostic stages involved in ${dp}$; ${s}_{i}$ indicates the $i{\rm{th}}$ diagnostic stage of ${dp}$, ${{DS}}_{i}$ represents the pathology knowledge involved in this stage;
- $${{DS}}_{i}=(\{{s}_{i}\}\cup {D}_{i}\cup {P}_{i},{R}_{{pd}}^{i}\cup {R}_{{sd}}^{i})$$, where
- ${D}_{i}=\{{D}_{i}^{+},{D}_{i}^{-}\}$ indicates the set of diagnostic possibilities considered by the pathologist at the $i{\rm{th}}$ stage, divided into the established outcomes ${D}_{i}^{+}$ and the excluded possibilities ${D}_{i}^{-}$;
- ${P}_{i}$ indicates the phenotypes the pathologist uses for diagnosing a patient;
- ${R}_{{pd}}^{i}\subseteq {P}_{i}\times {D}_{i}$ denotes the logical relationships between diagnostic features (phenotypes) and outcomes, including both supportive and contradictive correlations;
- ${R}_{{sd}}^{i}\subseteq \{{s}_{i}\}\times {D}_{i}$ indicates the corresponding relationship between the diagnostic stage and the diagnostic possibilities a pathologist considers within this stage.
- ${R}_{{ss}}\subseteq {S}_{{dp}}\times {S}_{{dp}}$ indicates the sequential order among diagnostic stages.
- ${R}_{{ds}}\subseteq dp\times {S}_{{dp}}$ indicates that the group of diagnostic stages are involved in ${dp}$.

A schematic overview of ${{\mathscr{G}}}_{{dp}}$ is provided in Supplementary Figure 1c.

### Pathology mark-up language

To standardize the creation of PathoGraph representations, we further develop PathoGraph into a markup language based on Web Ontology Language (OWL), known as Pathology Markup Language (PathoML). In PathoML, nodes of PathoGraph are implemented as either OWL classes or data properties, while edges are represented using object properties. This approach allows PathoGraph representations to be instantiated via PathoML elements, transforming the graph representations into standardized, machine-readable documents. As a result, PathoML facilitates the storage, exchange, and interpretation of pathology knowledge in accordance with the FAIR principles, enhancing the use of PathoGraph in diverse scientific and clinical contexts.

**Fig. 3:**
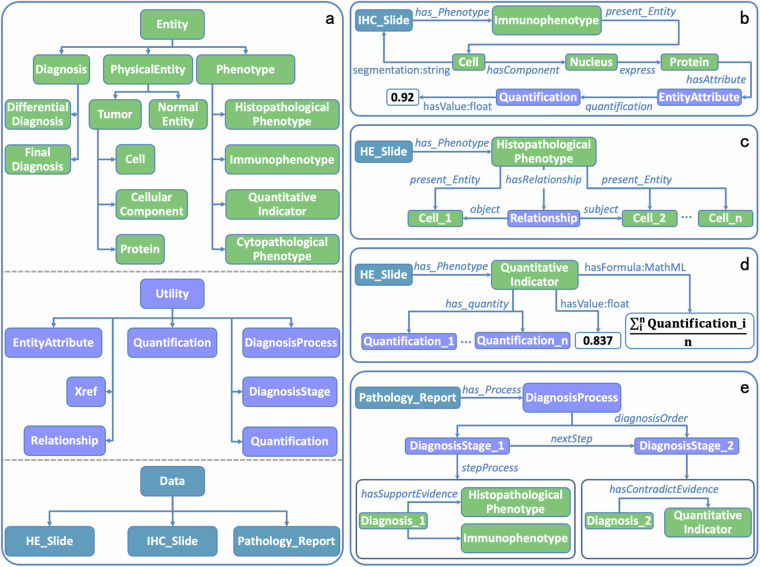
Overview of PathoML. **(a)** The high-level structure of PathoML in which PathoML classes are shown as boxes and arrows represent subclass relationships. (**b**–**e**) Illustration of how PathoML represents a single-cell immunophenotype, a multi-cell histopathological phenotype, a quantitative indicator and a diagnostic process, respectively. The individuals of PathoML classes are shown as boxes while the properties are shown as labelled arrows. The labels on the arrows indicating the object properties are italicized while the ones indicating the datatype properties are not. The labels of datatype properties are formed as “Name:Datatype”.

The architecture of PathoML classes is shown as Fig. 3a. **Entity,** **Utility** and **Data** are the three root classes. **Entity** includes classes regarding pathological entities, phenotypes and diagnoses, while **Utility** includes classes for pathological features, quantitative parameters, among others. **Data** includes classes for storing metadata of pathology data and PathoGraph representations. In the following sections, we describe PathoML classes and properties related to each subgraph of PathoGraph. The complete structures of **Entity,** **Utility**, and **Data** in PathoML are illustrated in Supplementary Figures 2, 3, and 4, respectively. A detailed explanation of PathoML is available in its language specification (see “Data Availability”).

#### PathoML elements related to pathology entity graph

The nodes of Pathology Entity Graph, including pathological entity, pathological feature, and quantitative parameter, are implemented in PathoML as **PhysicalEntity,** **EntityAttribute**, and **Quantification** respectively. To increase the specificity of **PhysicalEntity**, it is further divided into several subclasses based on biological scales, including **Cell,** **Cellular_Component,** **Protein** and others. The OWL properties related to Pathology Entity Graph are shown in Fig. 3b. The part-whole relationships among the entities are implemented as the object property *hasComponent*, and the associations between proteins and their residing cellular components are represented using *express*. Furthermore, *hasAttribute* is employed to link pathological entities with their distinct features, while relationships between pathological features and their quantitative parameters are defined using *quantification*. The datatype property ‘hasValue’ is designated for recording the numerical value of each quantitative parameter. Additionally, the ‘segmentation’ property serves to pinpoint the precise location of a pathological entity on the pathological slide.

#### PathoML elements related to pathology phenotype graph

PathoML employs **Phenotype** class to encapsulate various pathological phenotypes. This class is further subdivided into four distinct subclasses: **Cytopathological_Phenotype,** **Histopathological_Phenotype,** **Immunophenotype**, and **Quantitative_Indicators**.

**Cytopathological_Phenotype** and **Histopathological_Phenotype** describe single-cell and multi-cell phenotypes observed in H&E slides, whereas **Immunophenotype** pertains to those identified in IHC slides. The associated OWL properties are shown in Fig. 3c. *present_Entity* links each phenotype to one or more entities that constitute it. Additionally, PathoML introduces **Relationship** to articulate both spatial arrangement relationships and dynamic interactions among pathological entities involved in multi-cell phenotypes. **Relationship** encompasses two object properties: *subject* and *object*. In the context of dynamic behavior, *subject* links to the entity initiating the action (e.g. a tumor invading a renal sinus), whereas *object* links to the entity at which the action is directed (e.g. the renal sinus being invaded). Conversely, in depicting spatial arrangement, *subject* connects to the entity that acts as the reference point (e.g. a tumor enveloping a fibrovascular core), while *object* connects to the entity whose position or orientation is defined in relation to the subject entity (e.g the fibrovascular core being enveloped). Furthermore, **Relationship** could accommodate multiple instances of *subject* and *object* properties to represent complex relationships.

**Quantitative_Indicator** is designated to characterize quantitative phenotypic indicators. Its related properties are shown in Fig. 3d. *has_quantity* establishes a link between an instance of **Quantitative_Indicator** and the set of quantitative parameters involved in its computation. Furthermore, **Quantitative_Indicator** is equipped with two essential datatype properties: ‘hasValue’, which records the numerical outcome of the indicator, and ‘hasFormula’, which captures the computation formula in the standardized MathML format^10^.

#### PathoML elements related to pathology diagnosis graph

The nodes of Pathology Diagnosis Graph, such as diagnostic process, stage, and possibility, are represented as **DiagnosisProcess,** **DiagnosisStage**, and **Diagnosis**, respectively. Figure 3e depicts their properties. The property *diagnosisOrder* connects a **DiagnosisProcess** instance with its associated **DiagnosisStage** instances, indicating the stages within that process. *nextStep* links sequential **DiagnosisStage** instances, showing their progression through the diagnostic process. *stepProcess* associates a **DiagnosisStage** instance with one or more **Diagnosis** instances, representing the diagnostic possibilities considered at that stage. *hasSupportEvidence* and *hasContradictEvidence* properties define the supportive and contradictory connections between diagnostic features and diagnostic possibilities. Additionally, **Diagnosis** is categorized into **Differential_Diagnosis** and **Final_Diagnosis**, denoting the excluded possibilities and the diagnostic outcome respectively.

#### Other PathoML elements

In addition to PathoGraph’s elements, PathoML includes other OWL constructs designed to store metadata related to PathoGraph representations and associated pathology data. For example, **Data** includes **HE_slide,** **IHC_Slide** and **Pathology_Report** for storing metadata of pathological slides (e.g. height, width and magnification of a slide) and pathology reports respectively. *has_Phenotype* (Fig. 3b,c,d) and *has_Process* (Fig. 3e) link pathology data to PathoGraph representations of the pathology knowledge contained in the data. Additionally, PathoML provides **Xref** class for mapping PathoGraph representations to controlled vocabularies for achieving naming consistency, linked by the property *hasXref*.

### Exemplar use of PathoGraph for knowledge representation

To illustrate that PathoGraph could effectively represent the complex meaning of pathology knowledge, we provide several examples covering a single-cell immunophenotype, a multi-cell histopathological phenotype, and two diagnostic processes of pathologists.

#### HER2 immunophenotype of breast cancer

**Fig. 4:**
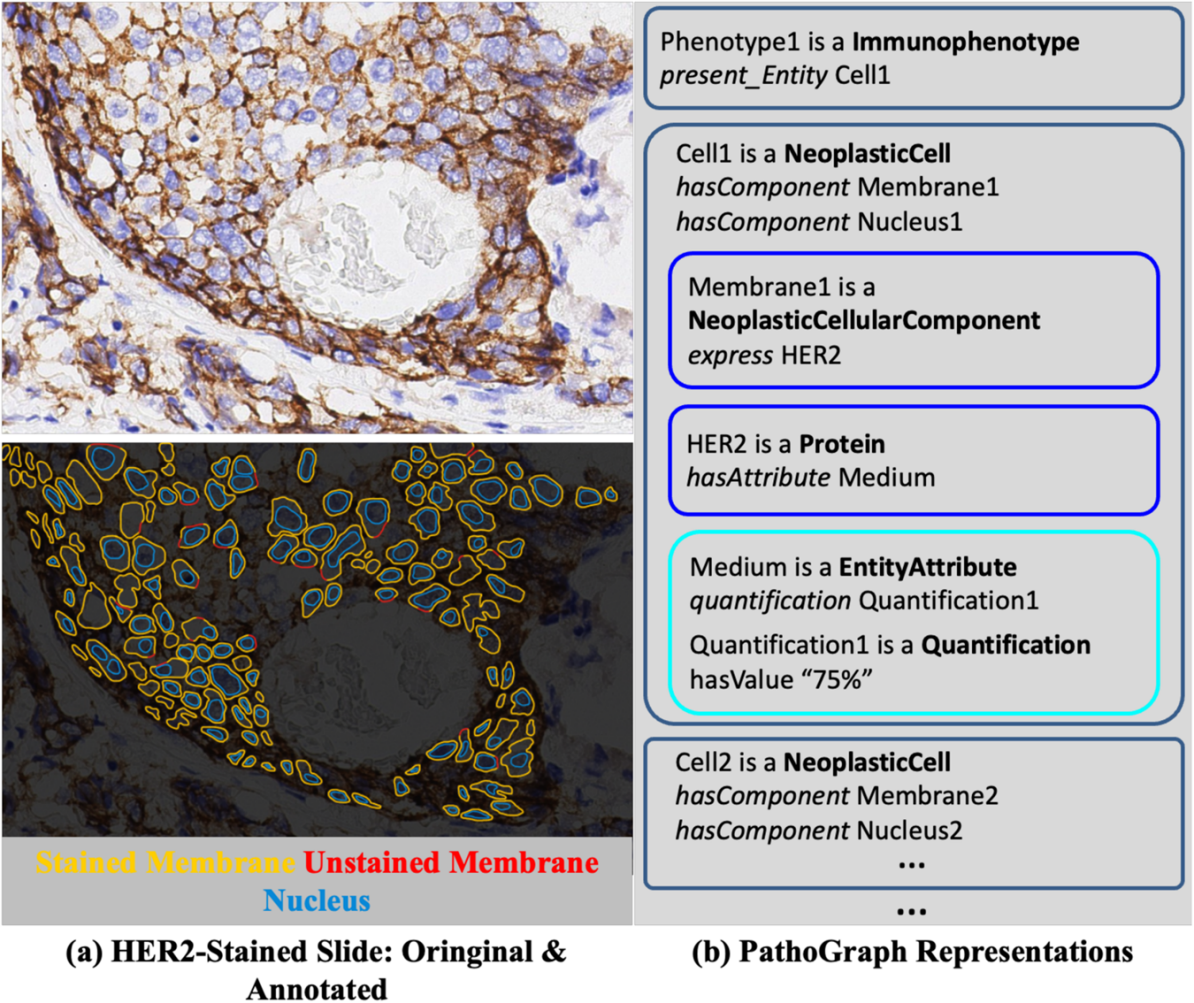
The exemplar PathoGraph representation of a HER2 Immunophenotype, presented in a form of PathoML. An individual of PathoML class is shown as a rounded rectangle. PathoML class names are highlighted in bold, the object properties are italicized while the datatype properties are not. “is a” indicates the declaration of an individual as belonging to a specific PathoML class.

We first select a breast cancer IHC slide labelled for HER2 antigen and use PathoGraph to describe the staining characteristics of the tumor cells in the slide, as shown in Fig. 4. Each tumor cell in the slide stained by HER2 indicates a single-cell immunophenotype. HER2 typically stains the cell membrane of a breast cancer tumor cell. More specifically, parts of the membrane where HER2 resides are stained, whereas other parts lacking HER2 remain unstained. Therefore, the extent to which the membrane is stained indicates the quantity of HER2 present in a tumor cell. The annotations in Fig. 4a specifically illustrate the stained and unstained parts of the cell membrane for each tumor cell.

As demonstrated in Fig. 4b, PathoGraph describes a HER2 immunophenotype as an instance of **Immunophenotype**, named ‘Phenotype1’. As for the HER2-stained tumor cell, it creates a **NeoplasticCell** instance named ‘Cell1’, referring to the tumor cell itself. Then, for the cell’s membrane and nucleus, it generates instances of **NeoplasticCellularComponent**, named ‘Membrane1’ and ‘Nucleus1’, respectively. Finally, an instance of **Protein**, named ‘HER2’, represents the HER2 antigen. ‘Cell1’ is connected to ‘Membrane1’ and ‘Nucleus1’ via *hasComponent*, and ‘Membrane1’ is linked to ‘HER2’ through *express*. To detail the staining characteristics, PathoGraph generates an **EntityAttribute** instance (named ‘Medium’) to represent the staining extent of the cell membrane. Additionally, a **Quantification** instance (named ‘Quantification1’) provides a quantitative measure of the staining extent, indicating that 75% of the cell membrane is stained (see “Methods” for details). The **EntityAttribute** and **Quantification** instances are associated through the *quantification* relationship. For brevity, the PathoGraph representations of other tumor cells have been omitted.

#### Follicular pattern of papillary thyroid carcinoma

**Fig. 5:**
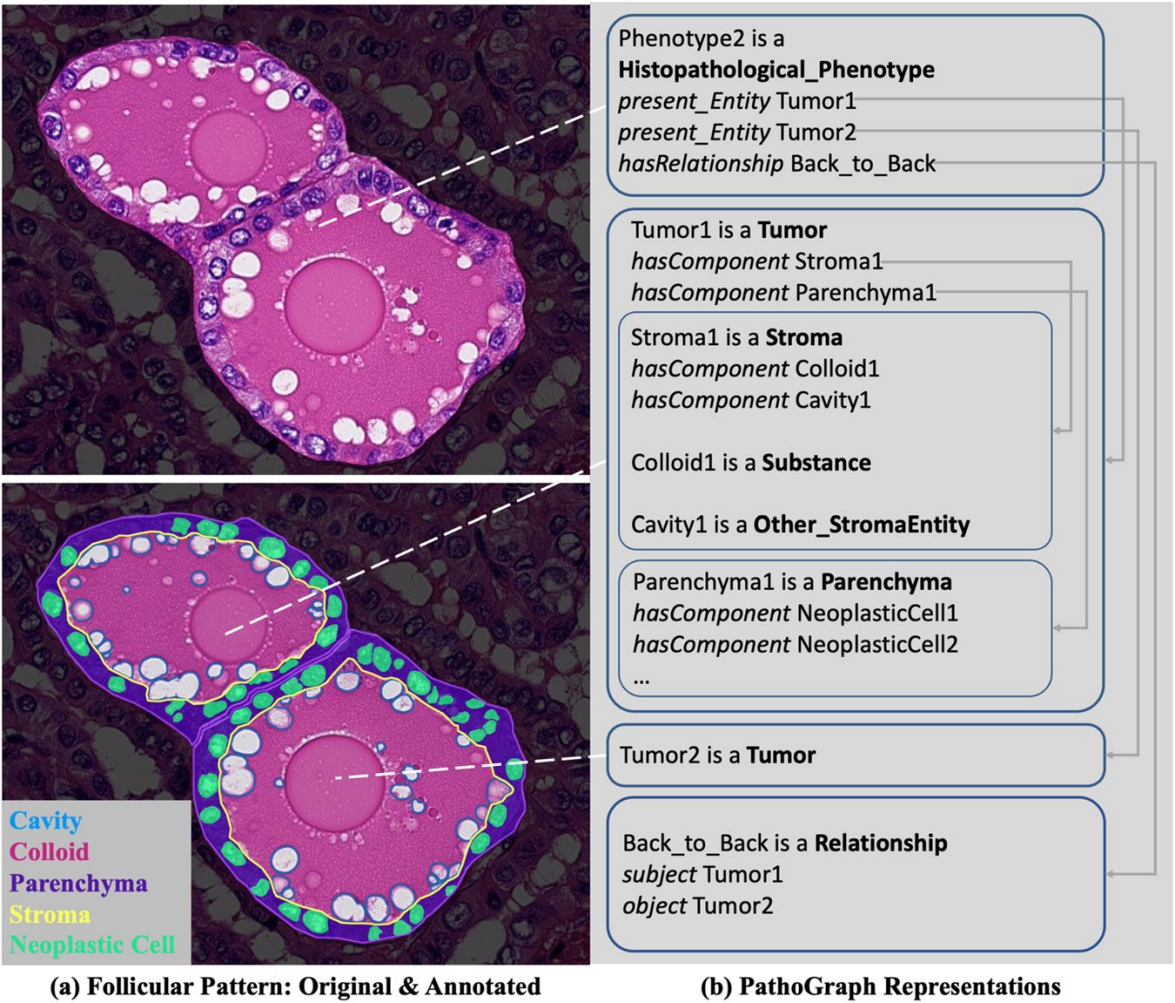
The exemplar PathoGraph representation of a follicular pattern.

We select a follicular pattern of papillary thyroid carcinoma to show how PathoGraph represents multi-cell phenotypes, as shown in Fig. 5. In this type of thyroid carcinoma, the normal follicular architecture of the thyroid gland is disrupted, leading to follicles being tightly packed together, sometimes with follicular structures pressing against each other. Figure 5a shows a back-to-back arrangement of two follicles, which is one of the important features in the pathological diagnosis of papillary thyroid carcinoma. PathoGraph describes this phenotype as an instance of **Histopathological_Phenotype** named ‘Phenotype2’. As for the follicles composing this phenotype, PathoGraph builds two instances of **Tumor** named ‘Tumor1’ and ‘Tumor2’ to indicate the follicles. Furthermore, it generates instances of **Stroma,** **Parenchyma,** **Substance** as well as **NeoplasticCell** to represent the follicles’ components, and links them together using *hasComponent*. In order to describe the back-to-back arrangement of these two follicles, we build a **Relationship** instance, wherein *subject* links to ‘Tumor1’ and *object* links to ‘Tumor2’.”

#### Histological subtyping process for a cervical pathological slide

**Fig. 6:**
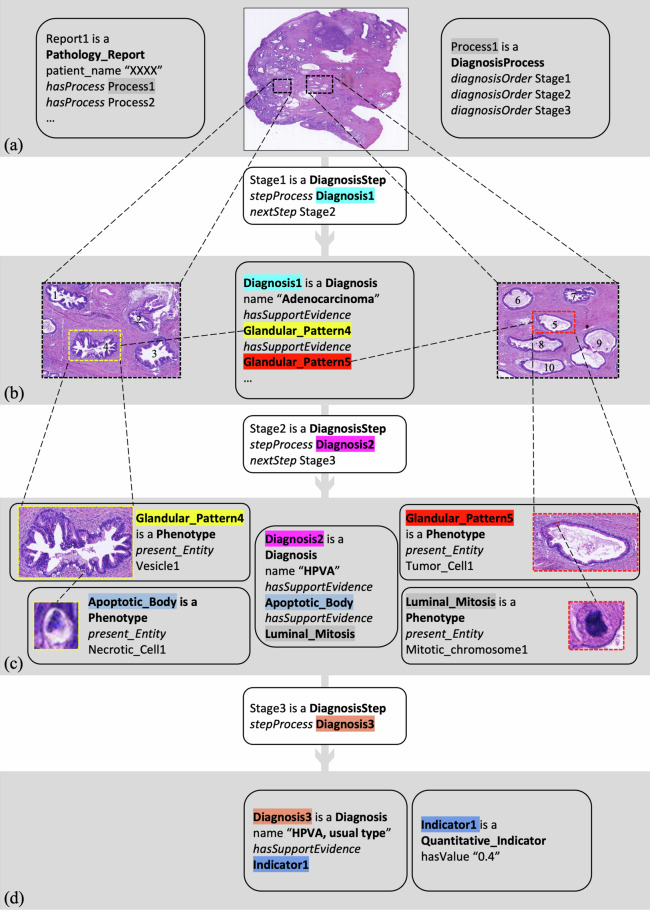
The exemplar PathoGraph representation of a histological subtyping process for a cervical pathological slide, presented in a form of PathoML. **(a)** An overview of the slide, and the PathoGraph representation of the diagnostic process, as well as the metadata of the corresponding pathology report from which this process is derived. (**b-d**) The PathoGraph representations of the three diagnostic stages involved in this diagnostic process.

Now we show how PathoGraph represents a diagnostic subtyping process of a pathologist for a cervical HE-stained pathological slide, as illustrated in Fig. 6. PathoGraph firstly creates a **DiagnosisProcess** instance (named ‘Process1’) indicating the subtyping process (Fig. 6a). This process consists of three individual diagnostic stages, represented by three **DiagnosisStage** individuals (named ‘Stage1’, ‘Stage2’, ‘Stage3’), to which *diagnosisOrder* links ‘Process1’. *nextStep* links these three **DiagnosisStage** individuals, showing their sequential order. The descriptions of diagnostic outcomes within each stage are as follows:

At the first stage, the pathologist identifies several widely-distributed glandular patterns under a microscope at low magnification, which leads to an initial diagnosis that this tumor is an adenocarcinoma. To describe this diagnosis, PathoGraph creates a **Diagnosis** instance (named ‘Diagnosis1’), and links it to the **Phenotype** instances describing those evidential glandular patterns via *hasSupportEvidence*, which is shown in Fig. 6b. Additionally, PathoGraph links ‘Stage1’ to ‘Diagnosis1’ through *stepProcess*.

Then, the pathologist performs a more detailed examination on the glandular patterns at medium and high magnification. In the two of glandular patterns, he detects apoptotic bodies (i.e. a vesicle containing parts of a dying cell) and luminal mitoses (i.e. a tumor cell with a mitotic chromosome close to the inner side of the gland). With these phenotypes in mind, he could further sub-categorize this patient as Human papillomavirus-associated endocervical adenocarcinoma (HPVA). Correspondingly, PathoGraph builds ‘Diagnosis2’ along with other relationships, as Fig. 6c shows.

Finally, the pathologist evaluates the tumor cells with intracytoplasmic mucin in the tumor areas and determines that 40% of the tumor cells contain intracytoplasmic mucin. This finding leads to the conclusion that the histologic type of the tumor is HPVA, usual type. PathoGraph represents this diagnosis using ‘Diagnosis3’. Additionally, it considers the proportion of tumor cells with intracytoplasmic mucin as a quantitative phenotypic indicator, which is represented through a **Quantitative_Indicators** instance, shown in Fig. 6d.

#### Differential diagnosis for histological subtyping of lymphoma

**Fig. 7:**
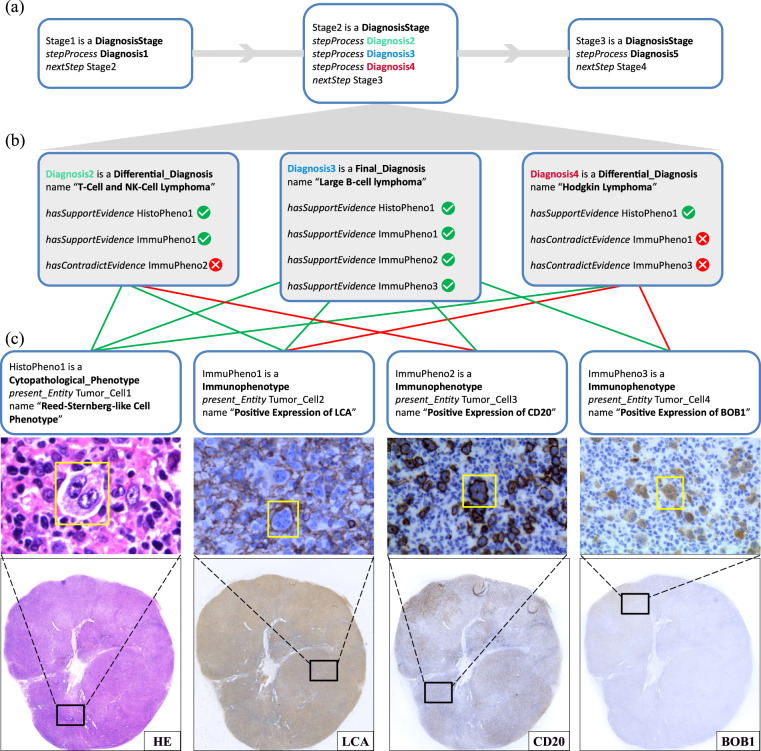
The exemplar PathoGraph representation of a differential diagnosis for histological subtyping of lymphoma, presented in a form of PathoML. **(a)** The PathoGraph representations of the three diagnostic stages. **(b)** The representations of the three diagnostic possibilities to be differentiated in the second stage. **(c)** The evidential phenotypes the pathologist uses to differentiate the diagnostic possibilities. Green lines represent supportive relationships and red lines represent contradictions between phenotypes and diagnostic possibilities.

We further demonstrate how PathoGraph represents a differential diagnostic process, as illustrated in Fig. 7. In this case, the pathologist differentiates among three possible lymphoma subtypes to ascertain the most accurate diagnosis. Initially, the pathologist evaluates the HE slide and makes a preliminary diagnosis of lymphoma. In the second stage, to refine the diagnosis, the pathologist differentiates among three subtypes of lymphoma, including T-Cell and NK-Cell lymphoma, large B-cell lymphoma, and Hodgkin lymphoma. Due to the insufficiency of the HE slide alone for a conclusive diagnosis, additional IHC testing results on LCA, CD20, and BOB1 are incorporated for further differentiation. The IHC results support large B-cell lymphoma and contradict the other subtypes, leading to its establishment as the diagnostic outcome at the second stage.

As Fig. 7a shows, although the pathologist could further differentiate subtypes of large B-cell lymphoma in the next stages, we mainly show PathoGraph represents the second stage of this diagnostic process. PathoGraph builds **Phenotype** instances to represent evidential phenotypes, including the one observed in the HE slide, and three other immunophenotypes, as Fig. 7c shows. Additionally, PathoGraph constructs **Differential_Diagnosis** instances for the excluded subtypes, and a **Final_Diagnosis** for the established subtype, linking them to the **Phenotype** instances through *hasSupportEvidence* and *hasContradictEvidence*, as Fig. 7b shows.

## Technical Validation

PathoGraph transforms pathology knowledge hidden and fragmented in pathology data into explicit and structured graph representations, thereby facilitating its computational use. To demonstrate this, we present a series of technical validations in this section.

### Automatic construction of pathology entity graph

A large-scale PathoGraph is essential for enabling AI-based pathology practices. To demonstrate the technical feasibility of automatically constructing PathoGraph, we take the Pathology Entity Graph (PEG) as an example and propose an automated workflow, which extracts pathological entities and their features from pathology image patches, assembles them into PEG, and ultimately translates the results into PathoML.

**Fig. 8:**
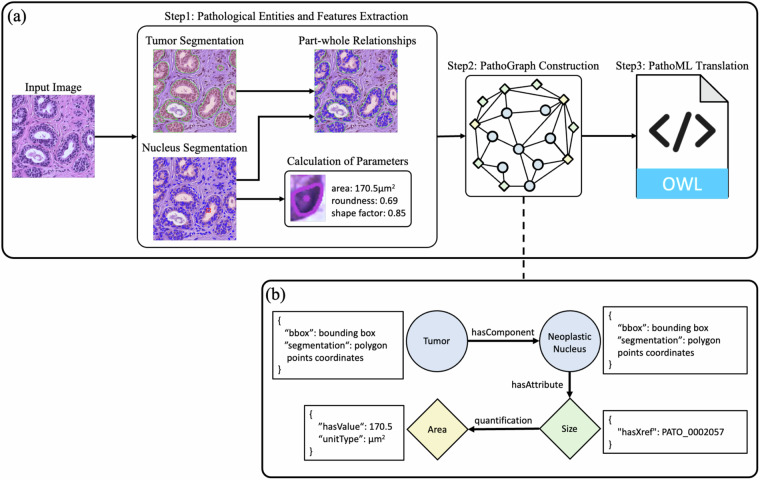
Overview of the automatic construction pipeline for a Pathology Entity Graph (PEG). **(a)** Pathological entities (tumors, nuclei) are segmented from the input image, their features (area, roundness, etc.) are computed, and part–whole relationships are established. **(b)** Schematic view of the generated PEG.

As shown in Fig. 8a, the automated construction of PEG consists of three steps: 1) Given a pathological image patch, we first segment the tumor regions using a tool named BiomedParse^11^, then detect both neoplastic and non-neoplastic nuclei via CellVit^12^. To capture the pathological features of neoplastic nuclei, we compute various morphological parameters, including area, roundness, and shape factor etc. Finally, we establish the part–whole relationships between the neoplastic nuclei and tumor regions based on their spatial overlap. 2) We treat the segmented tumors, neoplastic nuclei, and their morphological features as graph nodes, and encode the part–whole relationships between the tumors and their neoplastic nuclei, as well as the affiliations between the nuclei and their features, as graph edges. In this way, we generate a tumor PEG that describes the composition of each tumor cluster, as well as its morphologic characteristics. 3) Using the corresponding PathoML elements, all tumor PEGs extracted from the patch are collectively translated into PathoML and saved as a single OWL-format document.

We further construct a graph dataset by applying this workflow to the public BReAst Cancer Subtyping (BRACS) dataset^12^. For each patch image in BRACS—4,391 in total—we generate a corresponding PEG. As illustrated in Fig. 8b, each PEG uses nodes to represent the tumors and neoplastic nuclei recognized in the patch, with bounding box coordinates and segmentation outlines stored as node attributes. Additional nodes capture each neoplastic nucleus’s pathological features and quantitative parameters. The *hasComponent* edge links each tumor to its individual neoplastic nuclei, while *hasAttribute* and *quantification* edges connect a nucleus, its pathological features, and morphological parameters.

### Enhancing computational pathology model performance with PathoGraph

We choose patch-based subtyping as the learning task, to examine whether PathoGraph could improve deep learning-based computational pathology models. We continue using BRACS as the dataset, and the task is three-class classification, whose goal is to predict whether a given patch is benign tumors, atypical tumors or malignant.

Regarding the experimental design, we employ a GraphSAGE + SAGPooling framework^13^ for graph-based learning, which is widely used for embedding pathology patch images^14^, and MLP as the classifier. For each patch, we construct two distinct graph structures. The first one is constructed by splitting the patch into smaller sub-patches (or regions) and use a nearest-neighbor approach to connect these sub-patches as graph nodes, creating edges based on spatial proximity, while the second is PEG, as previously described. We then train the model separately on these two types of graphs, obtaining two sets of patch embeddings. Finally, we compare their performance on the downstream classification task. The details of the experiment setting are provided in ‘Methods’.

**Fig. 9:**
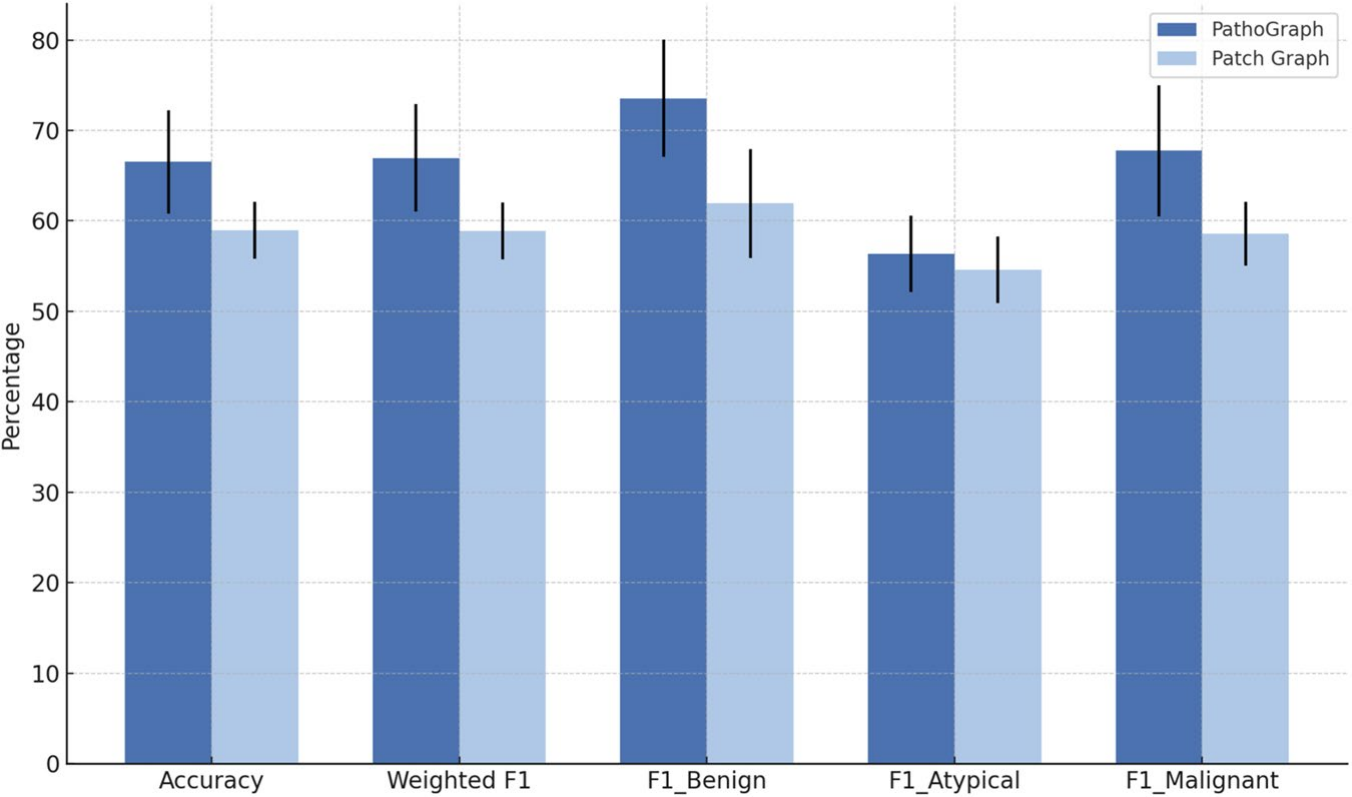
Performance comparison with mean and standard deviation. The bar chart shows the mean performance and standard deviation (represented by error bars) of two methods:one trained using PathoGraph and the other using patch graph, evaluated across five random seeds. Performance metrics include accuracy, weighted one-versus-rest F1 score, and one850versus-rest F1 scores for each class (“Benign”, “Atypical”, and “Malignant”).

As shown in Fig. 9, the model that incorporates PEG demonstrates superior performance, suggesting that PEG-based representations can indeed provide performance gains for downstream computational pathology tasks.

### Case study for clinical relevance of PathoGraph

To further establish PathoGraph’s clinical relevance, we present two illustrative use cases demonstrating how PathoGraph can support quantitative and traceable assessments in real-world diagnostic scenarios.

#### Quantitative analysis of HER2 expression

Accurate assessment of HER2 expression status in tumor cells is crucial for predicting the prognosis and treatment efficacy for breast cancer patients^15^. Pathology reports issued by hospitals typically provide only the assessment results of HER2 status, lacking details on the staining characteristics and methods used in the assessment. It not only diminishes the credibility of the results but also reduces their comparability for future research. PathoGraph representations of immunophenotypes encompass staining locations of antigens on tumor cells, as well as quantitative descriptions of the antigens’ staining characteristics. PathoGraph further allows for retrieval and analysis of these features. This could enable a quantitative and traceable assessment of the HER2 protein expression status in tumor cells.

**Fig. 10:**
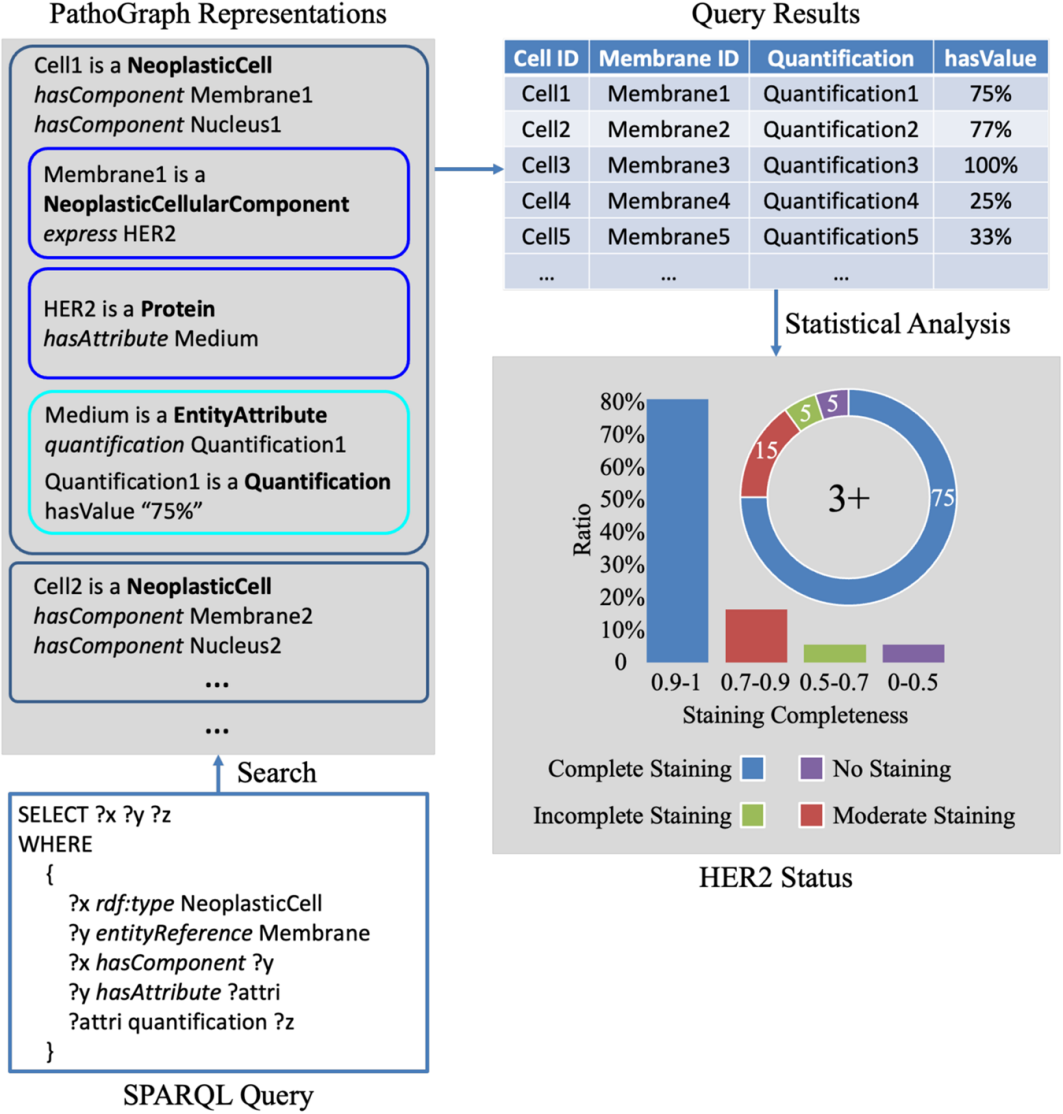
The workflow of quantitative analysis of HER2 expression.

We implement a use case of HER2 expression analysis based on the technique of SPARQL query. The workflow for this use case is depicted in Fig. 10. The PathoGraph representations being processed in this use case are previously illustrated in Fig. 4. Initially, the application constructs a SPARQL query using PathoML class names and property names as identifiers. This query retrieves tumor cells as well as their HER2 staining characteristics. The application then calculates the percentage distribution of staining extent across the membranes of tumor cells. Based on these quantitative results, the HER2 status is determined automatically based on the criteria outlined in Table 2. The distribution shows that 75% of tumor cells exhibit complete membrane staining, 15% show moderate completeness, 5% have incomplete staining, and 5% are unstained. Consequently, considering this percentage distribution, the HER2 protein expression status of this IHC slide is classified as ‘3 + ‘. Following this workflow, the HER2 status is determined based on quantitative metrics calculated using morphometric methods from pathology slides, rather than through visual observation of pathologists. Every step of the assessment as well as the features involved could be reviewed and verified, enhancing the credibility and reproducibility of the diagnostic results.

**Table 2:** The Assessment Criteria of HER2 Status.

HER2 Status	Assessment Criteria
3+	$>$10% tumor cells show complete staining (staining completeness $\in [0.9,\,1]$).
2+	$>$10% tumor cells show moderate staining (staining completeness $\in [0.7,\,9)$).
1+	$>$10% tumor cells show incomplete staining (staining completeness $\in [0.5,\,0.7)$).
0	>90% tumor cells are unstained (staining completeness $\in [0,\,0.5)$).

#### Automatic histologic subtyping

**Fig. 11:**
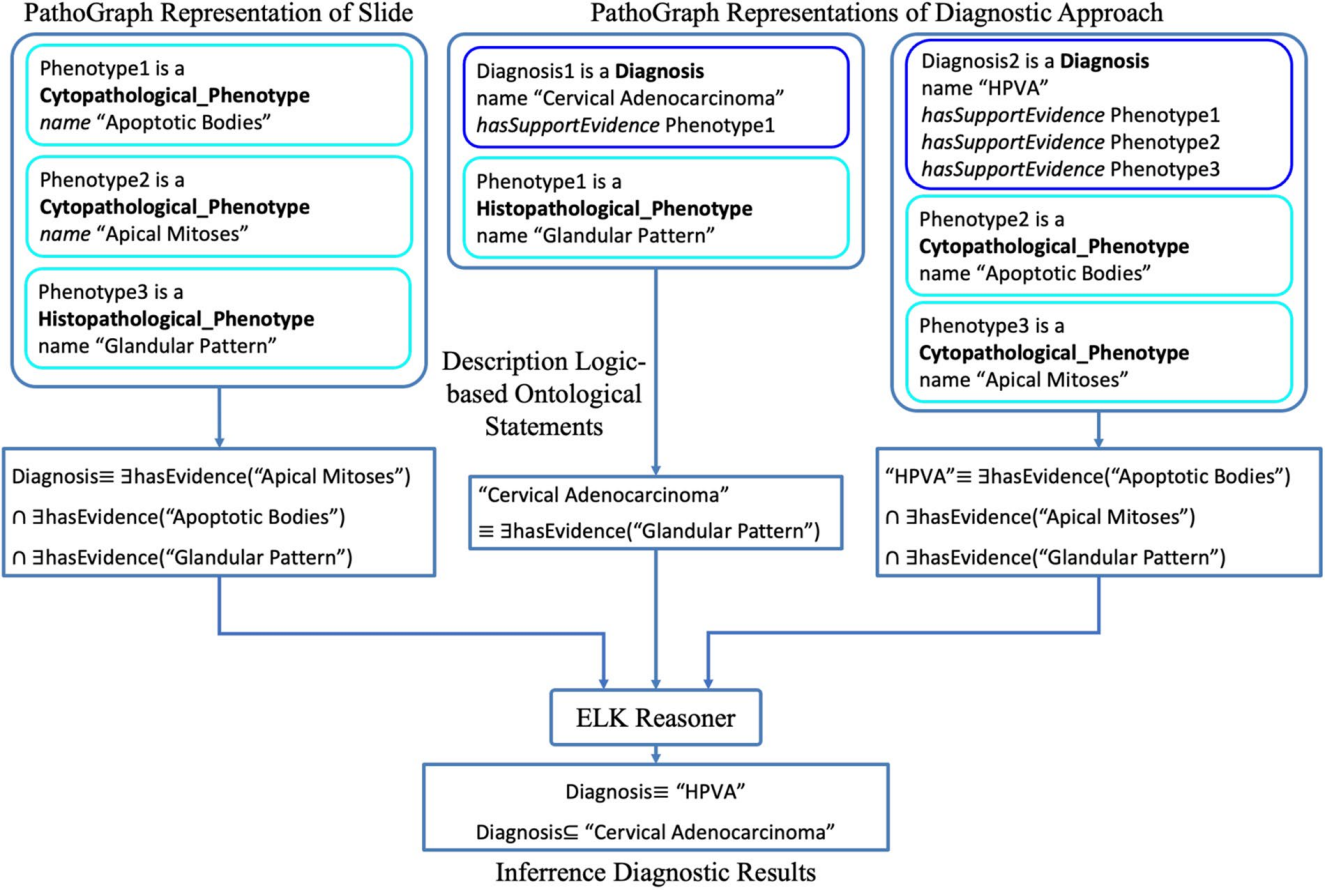
The workflow of automatic histologic subtyping. ∩ represents the conjunction operator, ≡ represents logical equivalence, ∃ represents the existential quantifier, ⊆ represents logical implication.

PathoGraph represents the diagnostic logics of pathologists in a comprehensive and structured way. Based on such representations, diagnostic assistance can be achieved automatically using various technical methods. To validate this application of PathoGraph, we implement a use case of automatic histologic subtyping based on the technique of ontological reasoning. The workflow for this use case is depicted in Fig. 11. The input of this workflow is a PathoGraph representation of phenotypes in a pathological slide, and a representation of a pathologist’s diagnostic approach used to diagnose the slide. The application then transforms the PathoGraph representations into ontological statements based on Description Logic^16^ (DL) respectively. It could infer a subtyping result by invoking an ontology reasoner (e.g. ELK^17^) to perform subsumption checking between the ontological statements.

Specifically, Fig. 11 shows a subtyping process for a cervical HE-stained pathological slide. The PathoGraph representation of this slide describes three pathological phenotypes observed within this slide: an apoptotic body, an apical mitosis and a glandular pattern. Additionally, the PathoGraph representation of the pathologist’s diagnostic approach, illustrated previously in Fig. 6, serves as the other input. These PathoGraph representations are then converted into ontological statements. Upon application of the ELK reasoner, it infers that the phenotypic evidence observed from the slide is logically implied under the ontological statement corresponding to cervical adenocarcinoma. Moreover, it establishes a logical equivalence between the statement of the slide and the one attributed to HPVA, indicating that the histological subtype of the slide is classified as HPVA.

## Discussion

In this section, we will discuss the significance of PathoGraph within the realm of AI-driven pathology practice. We firstly outline the applications of AI in the field of pathology and emphasize the importance of systematically organizing pathology knowledge contained in pathology data for these applications. Subsequently, we argue that PathoGraph would play a critical role in fulfilling this crucial yet unaddressed need, thus having potential to advance AI applications in disease research and clinical practice.

Recent advances in AI technology have spurred the development of new research domains in pathology. This includes computational oncology, deep phenotyping, and automated pathology diagnosis. In these domains, researchers employ statistical and machine learning methods to deeply analyze features derived from pathology data (referred to as “pathology knowledge” in this paper)^18^. This analysis helps in identifying patterns in the disease’s onset, progression, and diagnosis, shedding light on the mechanisms that cause diseases^19–21^, as well as assisting in their early detection and effective treatment^4,22–24^. Supplementary Table 7 details the objectives, methods, and key pathology knowledge underpinning computational analysis in these domains. However, a significant barrier to leveraging AI effectively in pathology is that pathology knowledge is hidden and fragmented across large datasets. Overcoming this hurdle is essential for actualizing the envisioned applications of AI in pathology.

This paper demonstrates that PathoGraph can not only represent pathology knowledge explicitly and comprehensively through its graph-based structure but also enhance its accessibility and computational utility. PathoGraph representations include detailed characteristics of diseases and encapsulate the diagnostic expertise of pathologists, potentially supporting AI analyses for disease understanding and automated diagnosis. Furthermore, the development of PathoML standardizes the creation, exchange, and integration of knowledge resources based on PathoGraph, paving the way for the construction of a robust pathology knowledge base. Much like how biomedical knowledge bases such as UniprotKB^25^, Reactome^26^, and BioModels^27^ have significantly advanced AI applications in biomedicine^28,29^, the integration of PathoGraph and PathoML is poised to establish a similar foundational framework for pathology. In this context, PathoGraph serves as the central graph model of the knowledge base, while PathoML drives consistent data exchange and interoperability. This graph-based knowledge base is anticipated to play a crucial role in developing scalable and robust AI methodologies for pathology.

An additional advantage of PathoGraph is its inherent capacity to fully leverage the strengths of graph machine learning^30^ (Graph ML), thereby improving the effectiveness of AI techniques in addressing real-world challenges in pathology. Graph ML excels in capturing complex semantics and hidden patterns within graph data, which are instrumental in addressing a range of downstream tasks. The application of Graph ML has already shown promising results in biomedicine and healthcare^31^, such as pinpointing genetic variants that contribute to complex traits, decoding single-cell behaviors, etc. Consequently, integrating PathoGraph with Graph ML could be a promising step towards unlocking the potential of AI in the field of pathology.

In conclusion, as the hidden and fragmented pathology knowledge impedes its computational utilization, we propose PathoGraph. It addresses the deficiency of a standardized knowledge representation method in the field of pathology, making a solid first step towards the goal of organizing and leveraging vast amounts of pathology knowledge in a highly efficient and intelligent manner. As AI technology advances, particularly with the advent of foundational models^32^, a vast amount of pathological knowledge could be automatically extracted from data and organized into PathoGraph representations. Given this fact, we believe that the value of PathoGraph will progressively elevate from a theoretical level to an application level, contributing to transformative developments in pathology.

## Methods

### Research process of PathoGraph

Making pathology knowledge contained in pathology data findable, accessible, interoperable, and reusable (FAIR) is crucial for AI applications in the field of pathology. To achieve this goal, we conducted a series of research endeavors over the past four years. Initially, we introduced the Histopathology Mark-up Language (HistoML)^33^, an OWL-based file format specifically designed for storing and exchanging annotations of digital pathology slides, notably Whole Slide Images (WSIs). Before the advent of HistoML, WSI annotations generated by different groups were stored in diverse file formats such as CSV, JSON, and XML, without a standardized or controlled vocabulary. This led to a significantly heterogeneous collection of annotated resources, that are extremely difficult to combine and reuse. HistoML was developed to standardize the exchange format and enhance the interoperability of WSI annotations.

However, as the application of AI in pathology continues to evolve, there is a clear shift from basic WSI analysis, which primarily focuses on the automatic extraction of image-based features, to more complex, context-rich tasks within biomedical and clinical disciplines. These advanced tasks, including computational oncology, deep phenotyping, and automatic diagnosis, require not only a deeper interpretation but also a broader integration of the comprehensive pathology knowledge contained within multi-modal pathology data. This need goes beyond simply analyzing a limited spectrum of cell and tissue types present in WSIs to generate meaningful insights.

Recognizing this need, along with the hidden and fragmented nature of pathology knowledge within pathology data, we develop PathoGraph. Unlike HistoML, PathoGraph is specifically designed to standardize the representation of knowledge in multi-modal pathology data, of which the content is much richer and more complex than WSI annotations. The aim of PathoGraph is to transform pathology data into a unified and computable knowledge framework, fostering more integrated, knowledge-driven AI applications in the field of pathology. Building on PathoGraph, we further refine HistoML and introduce PathoML, serving as the exchange and storage format for PathoGraph representations. The synergy between the knowledge representation method and the exchange format paves the way to make pathology knowledge FAIR.

### PathoGraph and PathoML development

Modelling knowledge in a graph structure is an effective and widely-used method for making knowledge computable, and graph representations of biomedical knowledge are prevalent in biomedical knowledge bases. For instance, UniProtKB utilizes a graph structure to describe protein knowledge^34^, where nodes represent proteins and their features, such as catalytic activity, physicochemical properties, and single nucleotide polymorphisms, and edges represent their interaction relationships. Similarly, Reactome^35^ and BioModels^36^ adopt graph structures to describe biological pathways, physiological processes, and drug actions by defining multiple sub-graphs. Given the proven efficacy of graph structures in representing intricate biomedical knowledge, a graph-based methodology is particularly well-suited for encapsulating the multifaceted and complex nature of pathology knowledge.

The development of PathoGraph and PathoML was conducted under the supervision and guidance of pathologists and medical professionals. The expert panel includes directors from the pathology departments of four grade “A” hospitals in China and the Mayo Clinic in the USA. The directors and their teams boast over 30 and 10 years of experience, respectively. In addition to the expertise of these pathology specialists, the development was also based on authoritative pathology textbooks^37,38^, diagnostic monographs^39^, and guidelines^8,40–43^, ensuring that PathoGraph definitions are rigorously grounded in knowledge.

### Implementation details for the pathograph performance experiments

We employ a three-stage Graph Neural Network (GNN) pipeline, where each stage consists of a GraphSAGE convolution layer followed by a self-attention pooling operation (SAGPooling). Specifically, we have three consecutive blocks, each applying GraphSAGE for feature aggregation and then SAGPooling to reduce the graph. After these three blocks, the pooled output is passed through a three-layer MLP for final classification. We adopt most hyperparameters from the paper^14^, except for the learning rate, which is set to 2 $\times$ 10^−5^.

For each patch, we construct two distinct graph structures which are later fed into the model: one is PEG, and the other is constructed by the nearest-neighbor approach. Specifically, the nearest-neighbor approach first crops the original patch into 256-pixel × 256-pixel regions and extracts the center point of each cropped patch. Then, for each small patch, it identifies k nearest neighbors and constructs edges between them. Finally, all small patch graphs are aggregated to form a complete graph.

Node embedding initialization is carried out by CONCH^44^. In the Pathology Entity Graph (PEG), each node corresponds to either the original patch, a tumor region, or a neoplastic nucleus. For a neoplastic nucleus, we extract a 512-dimensional embedding from its segmented region using CONCH, then append five morphological-feature parameters (e.g., shape factor, area etc.), yielding a total of 517 dimensions. For the tumor region and the original patch, we obtain 512-dimensional embeddings from CONCH and pad them with −1 to maintain the same 517-dimensional shape. As for the nearest-neighbor graph, each node is directly initialized with a 512-dimensional embedding from CONCH.

We select BRACS as the benchmark dataset and follow its original data split, where 60% is used as the training set, 20% as the validation set, and 20% as the hold-out testing set. The trained model which achieves highest validation accuracy is used for testing.

### Development of the exemplar representations and the use cases

The pathology data used to construct the exemplar PathoGraph representations were obtained from the First Affiliated Hospital of Xi’an Jiaotong University. Ethical review and approval of the study was provided by the hospital, and the reference number is KYLLSL-2021-420. Informed consent had been waived before the research was carried out. The data of the patients included in the study were de-identified and do not contain any protected health information or label text.

The staining extent of the cell’s membrane provided by Fig. 4b is calculated as follows:

$${\rm{Staining\; Extent}}={\rm{\theta }}/2{\rm{\pi }}$$

where θ represents the arc corresponding to the part of the cell membrane stained with the HER2 antigen. We used scikit-image^45^ libraries to calculate the staining completeness.

## Supplementary information

Supplementary Materials

## Data Availability

The graph dataset, the exemplar PathoML representations and the ontology specification of PathoML are freely available at https://github.com/Peiliang/PathoML.

## Code availability

The source code of the construction pipeline, the performance experiment and the two use cases are freely available at https://github.com/Peiliang/PathoML.

## References

[1] Boehm, K. M., Khosravi, P., Vanguri, R., Gao, J. & Shah, S. P. Harnessing multimodal data integration to advance precision oncology. *Nat. Rev. Cancer* **22**, 114–126 (2022). 34663944 10.1038/s41568-021-00408-3 PMC8810682

[2] Delude, C. M. Deep phenotyping: The details of disease. *Nature* **527**, S14–S15 (2015). 26536218 10.1038/527S14a

[3] Gurovich, Y. *et al*. Identifying facial phenotypes of genetic disorders using deep learning. *Nat. Med.* **25**, 60–64 (2019). 30617323 10.1038/s41591-018-0279-0

[4] Lu, M. Y. *et al*. AI-based pathology predicts origins for cancers of unknown primary. *Nature* **594**, 106–110 (2021). 33953404 10.1038/s41586-021-03512-4

[5] Shmatko, A., Ghaffari Laleh, N., Gerstung, M. & Kather, J. N. Artificial intelligence in histopathology: enhancing cancer research and clinical oncology. *Nat. Cancer* **3**, 1026–1038 (2022). 36138135 10.1038/s43018-022-00436-4

[6] Wilkinson, M. D. *et al*. The FAIR Guiding Principles for scientific data management and stewardship. *Sci. Data* **3**, 1–9 (2016). 10.1038/sdata.2016.18 PMC4792175 26978244

[7] College of American Pathologists. Protocol for the Examination of Biopsy Specimens from Patients with Invasive Carcinoma of Renal Tubular Origin. https://documents.cap.org/protocols/Kidney.Bx_4.1.0.0.REL_CAPCP.pdf (2021).

[8] Delahunt, B. *et al*. The International Society of Urological Pathology (ISUP) grading system for renal cell carcinoma and other prognostic parameters. *Am. J. Surg. Pathol.* **37**, 1490–1504 (2013). 24025520 10.1097/PAS.0b013e318299f0fb

[9] Ficarra, V., Galfano, A., Mancini, M., Martignoni, G. & Artibani, W. TNM staging system for renal-cell carcinoma: current status and future perspectives. *Lancet Oncol.* **8**, 554–558 (2007). 17540307 10.1016/S1470-2045(07)70173-0

[10] W3C. Mathematical Markup Language (MathML) Version 4.0. https://www.w3.org/TR/mathml4/.

[11] Zhao, T. *et al*. A foundation model for joint segmentation, detection and recognition of biomedical objects across nine modalities. *Nat. Methods* **22**, 166–176 (2025). 39558098 10.1038/s41592-024-02499-w

[12] Hörst, F. *et al*. CellViT: Vision Transformers for precise cell segmentation and classification. *Med. Image Anal.* **94**, 103143 (2024). 38507894 10.1016/j.media.2024.103143

[13] Lee, J., Lee, I. & Kang, J. Self-attention graph pooling. in *International conference on machine learning* 3734–3743 (pmlr, 2019).

[14] Chen, R. J. *et al*. Pathomic Fusion: An Integrated Framework for Fusing Histopathology and Genomic Features for Cancer Diagnosis and Prognosis. *IEEE Trans. Med. Imaging* **41**, 757–770 (2022). 32881682 10.1109/TMI.2020.3021387 PMC10339462

[15] Harris, L. *et al*. American Society of Clinical Oncology 2007 update of recommendations for the use of tumor markers in breast cancer. *J. Clin. Oncol. Off. J. Am. Soc. Clin. Oncol.* **25**, 5287–5312 (2007). 10.1200/JCO.2007.14.2364 17954709

[16] Baader, F., Horrocks, I., Lutz, C. & Sattler, U. *Introduction to Description Logic*. (Cambridge University Press, 2017).

[17] Kazakov, Y., Krötzsch, M. & Simancik, F. ELK reasoner: architecture and evaluation. in *ORE* (Citeseer, 2012).

[18] Lipkova, J. *et al*. Artificial intelligence for multimodal data integration in oncology. *Cancer Cell* **40**, 1095–1110 (2022). 36220072 10.1016/j.ccell.2022.09.012 PMC10655164

[19] Desbois, M. *et al*. Integrated digital pathology and transcriptome analysis identifies molecular mediators of T-cell exclusion in ovarian cancer. *Nat. Commun.* **11**, 5583 (2020). 33149148 10.1038/s41467-020-19408-2 PMC7642433

[20] AbdulJabbar, K. *et al*. Geospatial immune variability illuminates differential evolution of lung adenocarcinoma. *Nat. Med.* **26**, 1054–1062 (2020). 32461698 10.1038/s41591-020-0900-x PMC7610840

[21] Failmezger, H. *et al*. Topological Tumor Graphs: a graph-based spatial model to infer stromal recruitment for immunosuppression in melanoma histology. *Cancer Res.* **80**, 1199–1209 (2020). 31874858 10.1158/0008-5472.CAN-19-2268 PMC7985597

[22] Zhang, Z. *et al*. Pathologist-level interpretable whole-slide cancer diagnosis with deep learning. *Nat. Mach. Intell.* **1**, 236–245 (2019).

[23] Li, W. *et al*. Dense anatomical annotation of slit-lamp images improves the performance of deep learning for the diagnosis of ophthalmic disorders. *Nat. Biomed. Eng.* **4**, 767–777 (2020). 32572198 10.1038/s41551-020-0577-y

[24] Liu, Y. *et al*. A deep learning system for differential diagnosis of skin diseases. *Nat. Med.* **26**, 900–908 (2020). 32424212 10.1038/s41591-020-0842-3

[25] UniProt: the universal protein knowledgebase in 2021. *Nucleic Acids Res*. **49**, D480–D489 (2021). 10.1093/nar/gkaa1100 PMC7778908 33237286

[26] Gillespie, M. *et al*. The reactome pathway knowledgebase 2022. *Nucleic Acids Res.* **50**, D687–D692 (2022). 34788843 10.1093/nar/gkab1028 PMC8689983

[27] Malik-Sheriff, R. S. *et al*. BioModels—15 years of sharing computational models in life science. *Nucleic Acids Res.* **48**, D407–D415 (2020). 31701150 10.1093/nar/gkz1055 PMC7145643

[28] Bileschi, M. L. *et al*. Using deep learning to annotate the protein universe. *Nat. Biotechnol.* **40**, 932–937 (2022). 35190689 10.1038/s41587-021-01179-w

[29] Jumper, J. *et al*. Highly accurate protein structure prediction with AlphaFold. *Nature* **596**, 583–589 (2021). 34265844 10.1038/s41586-021-03819-2 PMC8371605

[30] Xia, F. *et al*. Graph Learning: A Survey. *IEEE Trans. Artif. Intell.* **2**, 109–127 (2021).

[31] Li, M. M., Huang, K. & Zitnik, M. Graph representation learning in biomedicine and healthcare. *Nat. Biomed. Eng.* **6**, 1353–1369 (2022). 36316368 10.1038/s41551-022-00942-x PMC10699434

[32] Chen, R. J. *et al*. A general-purpose self-supervised model for computational pathology. ArXiv Prepr. ArXiv230815474 (2023).

[33] Lou, P. *et al*. HistoML, a markup language for representation and exchange of histopathological features in pathology images. *Sci. Data* **9**, 1–12 (2022). 35803960 10.1038/s41597-022-01505-0 PMC9270329

[34] UniProtKB. Uniprot Knowledge Base XML Schema. https://ftp.uniprot.org/pub/databases/uniprot/current_release/knowledgebase/complete/uniprot.xsd.

[35] Demir, E. *et al*. The BioPAX community standard for pathway data sharing. *Nat. Biotechnol.* **28**, 935–942 (2010). 20829833 10.1038/nbt.1666 PMC3001121

[36] Keating, S. M. *et al*. SBML Level 3: an extensible format for the exchange and reuse of biological models. *Mol. Syst. Biol.* **16**, e9110 (2020). 32845085 10.15252/msb.20199110 PMC8411907

[37] Molavi, D. W. *The Practice of Surgical Pathology: A Beginner’s Guide to the Diagnostic Process*. (Springer, 2017).

[38] 范嫏娣, 张宝麟 & 江昌新. 王德延肿瘤病理诊断学. (天津科学技术出版, 1998).

[39] MD, T. A. L. Mills and Sternberg’s Diagnostic S*urgical Pathology*. (LWW, Philadelphia, 2022).

[40] Moch, H. Female genital tumours: WHO Classification of Tumours, Volume 4. *WHO Classif. Tumours* 4 (2020).

[41] Moch, H., Cubilla, A. L., Humphrey, P. A., Reuter, V. E. & Ulbright, T. M. The 2016 WHO classification of tumours of the urinary system and male genital organs—part A: renal, penile, and testicular tumours. *Eur. Urol.* **70**, 93–105 (2016). 26935559 10.1016/j.eururo.2016.02.029

[42] Alaggio, R. *et al*. The 5th edition of the World Health Organization classification of haematolymphoid tumours: lymphoid neoplasms. *Leukemia* **36**, 1720–1748 (2022). 35732829 10.1038/s41375-022-01620-2 PMC9214472

[43] Tan, P. H. *et al*. The 2019 World Health Organization classification of tumours of the breast. *Histopathology* **77**, 181–185 (2020). 32056259 10.1111/his.14091

[44] Lu, M. Y. *et al*. A visual-language foundation model for computational pathology. *Nat. Med.* **30**, 863–874 (2024). 38504017 10.1038/s41591-024-02856-4 PMC11384335

[45] Van der Walt, S. *et al*. scikit-image: image processing in Python. *PeerJ* **2**, e453 (2014). 25024921 10.7717/peerj.453 PMC4081273

