# Supplementary material for: PathoGraph: A Graph-Based Method for Standardized Representation of Pathology Knowledge
Source: Sci Data. 2025 May 27;12:872. doi: 10.1038/s41597-025-04906-z (PMC12117166; doi:10.1038/s41597-025-04906-z)

Supplementary Table. 1 | **Examples of Pathological Entities and Their Hierarchical Composition**

Pathological Entities	Primary Components	Secondary Components
Thyroid Artery	Thyroid Artery Wall	Intima, Media, Adventitia, Endothelium, Elastic Fibers, etc.
	Thyroid Artery Lumen	Red Blood Cells, etc.
Tumor Tissue	Parenchyma	Tumor Cells
	Stroma	Capillaries, Tumor Basement Membrane, etc.
Tumor Cell	Membrane	Phospholipids, Cholesterol, Membrane Proteins, etc.
	Cytoplasm	Proteins, Lipids, Carbohydrates, Vacuoles, etc.
	Nucleus	Nuclear Membrane, Proteins, Chromatin, Nucleolus, etc.

Supplementary Table. 2 | **Pathological Features of Tumor Cells and Their Cellular Components**

Pathological Entities	Pathological Features
Tumor Cell	Size, Shape, Border Completeness, Proliferative Activity, etc.
Nucleus	Size, Shape, Intracellular Position, Nuclear Membrane Thickness, Quantity
Nucleolus	Microscopic Clarity, Texture, Quantity, etc.
Cytoplasm	Cytoplasmic Basophilia, Texture, etc.

Supplementary Table. 3 | **Examples of Quantitative Parameters**

Pathological Features	Quantitative Parameters
Quantity	Cell Count, Mitotic Count, Spot Optical Density, etc.
Shape	Curvature, Shape Factor, Roundness, Elliptical Eccentricity, etc.
Size	Length, Width, Area, Perimeter, etc.
Texture	Correlation, Directionality, Entropy, Contrast, Energy, etc.

Supplementary Table. 4 | **Examples of Single-Cell Phenotypes**

Single-Cell Phenotypes	Definitions
Rhabdoid Cell	The rhabdoid cells are large neoplastic cells with eosinophilic intracytoplasmic inclusions and their nuclei are irregularly-shaped and eccentric with prominent nucleoli
Tumor Giant Cell	The tumor giant cells are characterized by the presence of one or several abnormally enlarged nuclei, exhibiting significant pleomorphism with irregular nuclear shapes, coarse and uneven chromatin distribution. These cell types are commonly associated with highly malignant tumors, their presence indicating rapid tumor growth and a potent proliferative potential.
Tumor Cell with Nuclear Groove	Tumor cells with nuclear grooves exhibit invaginations of the nuclear membrane into the nucleolus, forming irregular foldings or groove-like structures.
Tumor Cell with Strong HER2 Expression	Tumor cells exhibit strong, complete, and uniform membrane staining for HER2, indicating the overexpression of the HER2 protein on their surface. Such phenotypes are commonly associated with increased invasiveness and proliferative activity of tumor tissues.

Supplementary Table. 5 | **Examples of Multi-Cell Phenotypes**

Multi-Cell Phenotypes	Definitions
Follicular Pattern	The follicular pattern is characterized by structures formed by clusters of tumor cells, with a center containing colloidal substances and potentially multiple cavities. This phenotype is commonly observed in certain lymph node tumors and specific types of thyroid cancer, exhibiting various forms including individual follicles, back-to-back follicles, etc.
Papillary Pattern	The papillary pattern is characterized by a peripheral layer of tumor cells and a centrally supporting fibrovascular core. This phenotype and its variants, such as micropapillary and pseudopapillary patterns, exhibit unique morphological features and are prevalent in certain tumors of organs like the breast, thyroid, pancreas, and kidney.
Tumor Compression and Invasion into the Renal Vein	At the interface between the tumor and the renal vein, dense and uniform fibrous tissue is observed. Tumor tissue adheres to the venous wall and is associated with thrombosis. Within the tumor, accumulation of fibrin and leukocytes is evident. The tumor partially envelops the renal vein, indicating a propensity for vascular invasion.

Supplementary Table. 6 | **Examples of Quantitative Phenotypic Indicators**

Quantitative Phenotypic Indicators	Definitions
Tumor-Stroma Ratio (TSR)[1]	TSR is determined by calculating the proportion of the total area of the tumor parenchyma (i.e., the entirety of tumor cells) to the total area of the tumor stroma in a Hematoxylin and Eosin-stained pathological slide. Commonly, a higher TSR (indicating a higher proportion of tumor cells) is associated with a more favorable prognosis, while a lower TSR (indicating a higher proportion of stromal cells) may suggest a worse prognosis.
Tumor-Infiltrating Lymphocytes (TILs)[2]	TILs is quantified by calculating the ratio of the total area occupied by lymphocytes within the tumor tissue to the total area of the tumor tissue in a Hematoxylin and Eosin-stained pathological slide. In various cancers, such as breast cancer, melanoma, and colorectal cancer, a higher TILs value is commonly associated with improved survival rates and a positive response to immunotherapy.
HER2 Status[3]	The HER2 status is assessed by evaluating the proportion of tumor cells displaying specific staining patterns on immunohistochemically stained slides. The scoring system categorizes the staining patterns as (0) no staining, (1+) incomplete or faint membrane staining, (2+) complete membrane staining with weak to moderate intensity, and (3+) complete, strong, and uniform membrane staining. Based on the proportion of cells with different staining patterns, the score ranges from 0 to 3+. A higher score indicates a higher content of HER2 protein in the patient's pathological tissue, which is associated with a likelihood of favorable response to HER2-targeted immunotherapy.

1. Souza da Silva, R. M. *et al.* Standardized Assessment of the Tumor-Stroma Ratio in Colorectal Cancer: Interobserver Validation and Reproducibility of a Potential Prognostic Factor. *Clin. Pathol. Thousand Oaks Ventura Cty. Calif* **14**, 2632010X21989686 (2021).
2. Salgado, R. *et al.* The evaluation of tumor-infiltrating lymphocytes (TILs) in breast cancer: recommendations by an International TILs Working Group 2014. *Ann. Oncol.* **26**, 259–271 (2015).
3. Yang Wentai & Bu Hong. Guidelines for HER2 Testing in Breast Cancer (2019 Edition). Chinese Journal of Pathology 48, 169-175 (2019).

Supplementary Table. 7 | **AI-driven Research Domains in Pathology and Their Need for Pathology Knowledge**

Research Directions	Research Objectives	Research Methods	Pathology Knowledge Required
Computational Oncology	Investigate the inherent mathematical laws in the tumor system and its evolutionary process to better understand the mechanisms of tumor pathogenesis.	Conduct mathematical modelling and simulation of tumor evolution, quantitatively analyze the spatiotemporal dynamics and interactions of its multi-scaled components.	The characteristics of changes in pathological entities composing tumor tissues, and the quantitative parameters for measuring the extent of their changes 。
Deep Phenotyping	Investigate the quantitative definitions of various pathological phenotypes to advance the study of phenomics and the precision of disease diagnosis.	Employ statistical analysis techniques to integrate and analyze multi-scale characteristics of phenotypes, thereby quantitatively and finely understanding pathological phenotypes	The composition of pathological phenotypes, the characteristics and quantitative parameters of their constitutive entities, their spatial arrangement, and biological behaviors
Automated Pathology Diagnosis	Develop a pathology AI diagnostic system with the capability of differential diagnosis across various diseases, thereby assisting pathologists in diagnosing challenging cases	Construct AI systems with interpretability, generalizability, and strong reasoning capabilities, by using knowledge-guided and data-driven ways in combination.	The diagnostic approaches for various diseases, such as evidential phenotypes, the interpretation of phenotypes, estimation of diagnostic possibilities, and the process of differential diagnosis.

Supplementary Figure. 1 | **The Schematic Overview of PathoGraph. (a)** $\mathcal{G}_a$ denotes the Pathology Entity Graph. **(b)** $\mathcal{G}_{sp}^*$ refers to the expanded graph representation of a single-cell phenotype, and $\mathcal{G}_{mp}^*$ refers to the expanded graph representation of a multi-cell phenotype. Both $\mathcal{G}_{sp}^*$ and $\mathcal{G}_{mp}^*$ include Pathology Entity Graphs shown in dark-blue rounded rectangle, denoted as $\mathcal{G}_{a_{sp}}$ and $\mathcal{G}_{a_i}$ (i=1,2,3,4). $\mathcal{G}_{qi}$ refers to a Quantitative Indicator Graph. **(c)** $\mathcal{G}_{dp}$ denotes the Pathology Diagnosis Graph, which contains Pathology Phenotype Graphs labeled as $\mathcal{G}_{sp_1}$, $\mathcal{G}_{mp_1}$ and $\mathcal{G}_{qi_1}$.

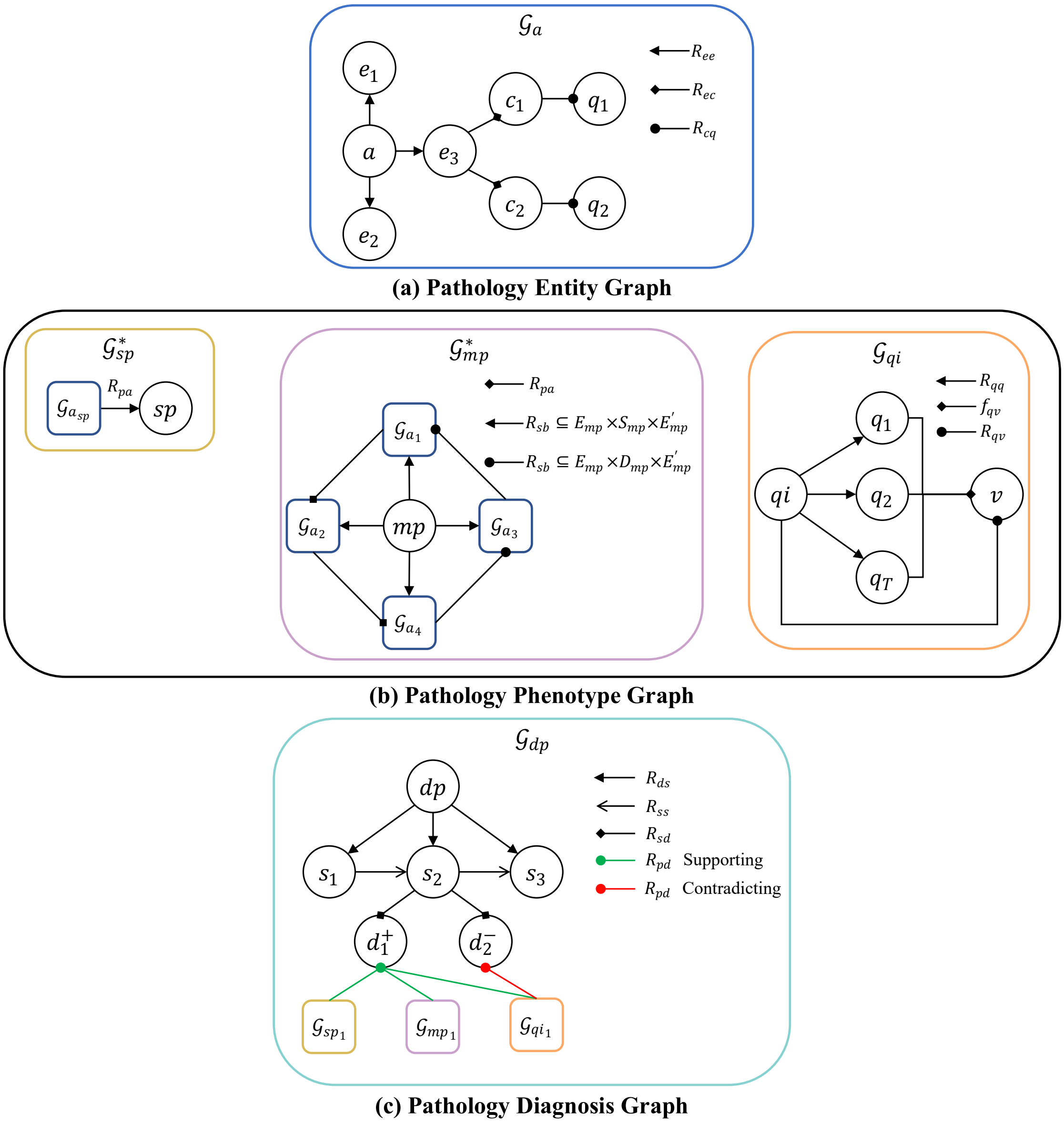

Supplementary Figure. 2 | **The Structure of Entity**

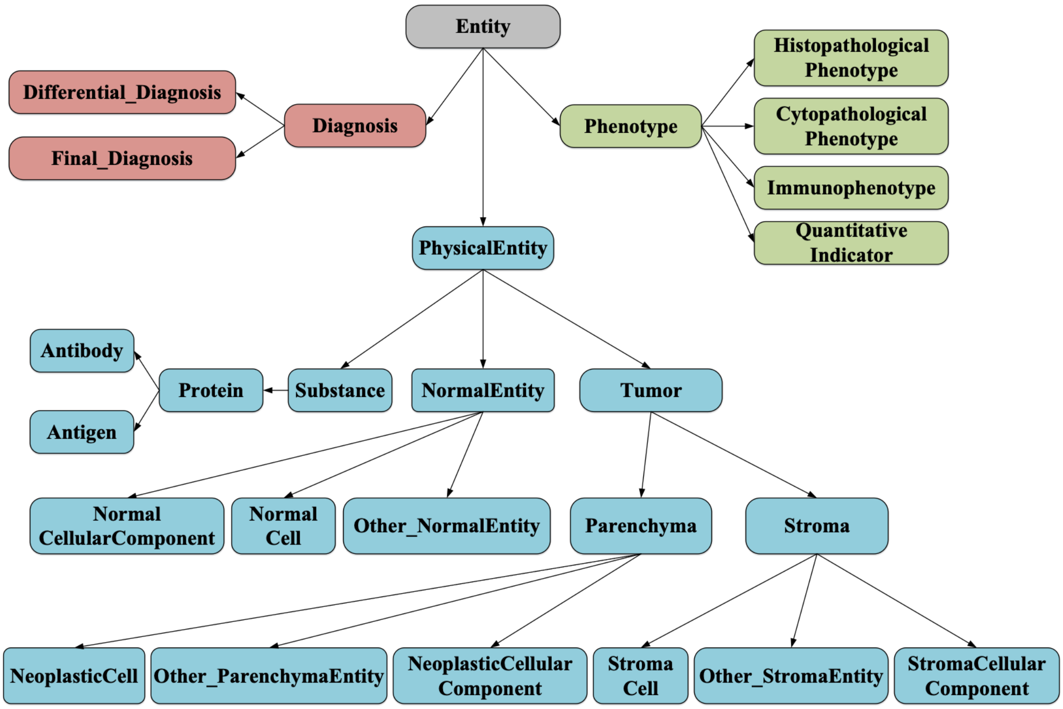

Supplementary Figure. 3 | **The Structure of Utility**

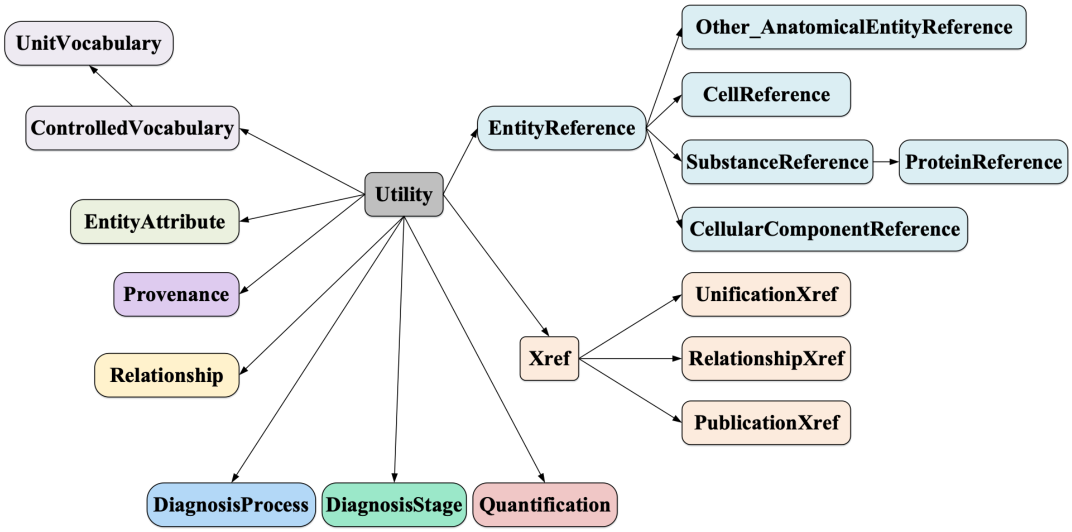

Supplementary Figure. 4 | **The Structure of Data**

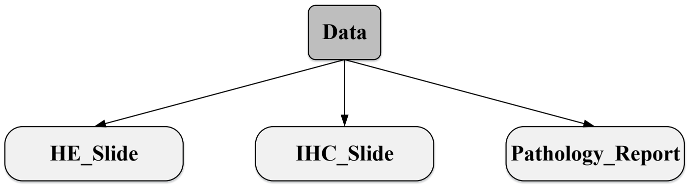

Supplement: Supplementary file 1 — Supplementary Materials [file 41597_2025_4906_MOESM1_ESM.pdf]
